# Butyrate protects against *Klebsiella pneumoniae*-induced oxidative stress in alveolar macrophages via p62-Keap1-Nrf2 pathway

**DOI:** 10.1016/j.redox.2026.104156

**Published:** 2026-04-12

**Authors:** Bao Meng, Hongru Li, Mingyang Tang, Jie Zhu, Ruixuan Yao, Ying Xu, Qingyue Zhang, Rongqing Zhu, Yaqin Guo, Yuexin Xu, Yuanlong Shu, Liang Yu, Yasheng Li, Yi Yang, Yanyan Liu, Ting Wu, Jiabin Li

**Affiliations:** a Department of Infectious Diseases & Anhui Center for Surveillance of Bacterial Resistance, The First Affiliated Hospital of Anhui Medical University, Hefei, 230022, China; b Anhui Province Key Laboratory of Infectious Diseases & Institute of Bacterial Resistance & Institute of Infectious Diseases, Anhui Medical University, Hefei, 230022, China; c Department of Neurology, The First Affiliated Hospital of Anhui Medical University, Hefei, 230022, China

**Keywords:** *Klebsiella pneumoniae*, Oxidative stress, Butyrate, p62, 4-HNE

## Abstract

*Klebsiella pneumoniae* (*K. pneumoniae*)-induced pneumonia poses growing clinical challenges due to the emergence of hypervirulent and carbapenem-resistant strains. Butyrate contributes to antibacterial immunity while its role in pulmonary infections remains poorly understood. Here, we showed that *K. pneumoniae* infection caused alveolar macrophage depletion and concomitant inflammatory tissue damage, while butyrate administration preserved alveolar macrophage populations and attenuated pulmonary damage. Mechanistically, butyrate upregulated both the expression and phosphorylation of p62, and regulated the Keap1-Nrf2 signaling pathway to counter oxidative stress. Moreover, we demonstrated that *K. pneumoniae* infection triggered oxidative stress injury in critically ill pneumonia patients. Circulating monocytes exhibited elevated levels of 4-hydroxynonenal (4-HNE), a marker of oxidative stress, along with downregulated mRNA levels of *SQSTM1* and *NRF2,* which were inversely correlated with 4-HNE levels. These findings establish butyrate as a dual modulator of the p62-Nrf2 antioxidant axis, highlighting its therapeutic potential for mitigating oxidative stress in *K. pneumoniae*-associated pneumonia.

## Highlights

• Butyrate rescues alveolar macrophages during *K. pneumoniae* pneumonia.
• Butyrate promotes p62-dependent autophagic degradation of Keap1 and nuclear translocation of Nrf2.
• Butyrate mitigates *K. pneumoniae*-induced macrophage apoptosis.
• Peripheral blood monocyte 4-HNE levels are elevated in patients with *K. pneumoniae* pneumonia.

## Graphical abstract

**Figure:**
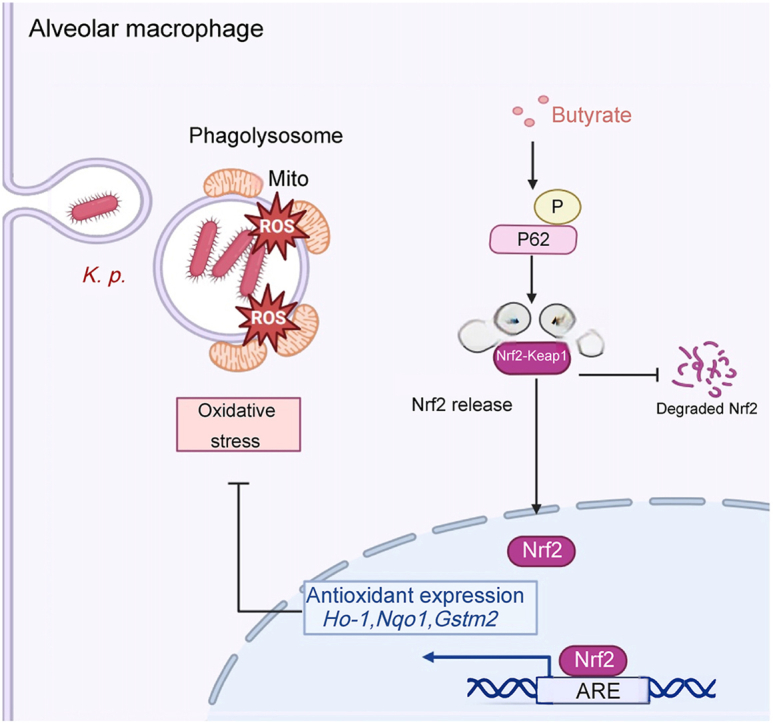

## 1 Introduction

*K. pneumoniae* is a Gram-negative bacterium with the ability to acquire new genetic traits and become either hypervirulent or antibiotic resistant, which renders treatment of the resulting infections increasingly difficult [[1], [2], [3]]. In particular, this pathogen causes severe infectious diseases, including pneumonia, liver abscess and sepsis, in both immunocompromised and otherwise healthy individuals, and has therefore emerged as a major clinical and public-health threat worldwide [4]. To address these challenges, it is important to develop innovative therapeutic strategies.

Upon stimulation and phagocytosis, macrophages generate large quantities of reactive oxygen species (ROS) through NADPH oxidase at the phagosomal membrane, a process essential for microbial killing [5,6]. However, excessive ROS accumulation provokes oxidative stress, leading to molecular damage-including DNA mutations, lipid peroxidation and protein oxidation-that contributes to cellular aging [5,7]. Therefore, maintaining cellular redox homeostasis is critical for the antimicrobial defense of macrophages. The transcription factor nuclear factor erythroid 2–related factor 2 (Nrf2) restores redox homeostasis by binding to enhancer sequences termed antioxidant response elements (AREs) and inducing a cluster of genes that encode antioxidant enzymes [7,8]. Beyond redox control, activated Nrf2 modulates macrophage inflammatory response by suppressing transcription of pro-inflammatory cytokines [9]. Kelch-like ECH-associated protein 1 (Keap1) is a negative regulator that targets Nrf2 for ubiquitination and proteasomal degradation under basal conditions [10]. During oxidative or electrophilic stress, electrophiles modify cysteine residues in Keap1, disabling its role as a substrate adaptor of E3-ligase and permitting rapid accumulation and nuclear translocation of Nrf2 [11].

Protein p62, also known as Sequestosome 1 (SQSTM1), contains several functional domains, including an light chain 3 (LC3)-interacting region (LIR), a Keap1-interacting region (KIR), and a ubiquitin-associated (UBA) domain, etc. These functional structural elements enable p62 to engage multiple signaling pathways that govern cellular metabolism, inflammation and death [12,13]. For instance, p62 acts as a selective autophagy receptor by binding ubiquitin-tagged cargo and LC3, thereby directing cytoplasmic components to lysosomal degradation [14]. Phosphorylation of p62 increases its binding affinity for Keap1, disrupts the Keap1-Nrf2 complex, and promotes nuclear translocation of Nrf2, indicating the crosstalk between autophagy and antioxidant signaling [[15], [16], [17]]. Transcription regulation and diverse post-translational modifications fine-tune p62 activity, and dysregulation of p62 is implicated in numerous diseases, including cancer, metabolic disorders and neurodegeneration [[18], [19], [20]].

Short-chain fatty acids (SCFAs) are produced by microbial fermentation of indigestible dietary carbohydrates in the proximal colon, where they are largely absorbed and contribute to intestinal mucosal immunity [21]. Emerging evidence suggests crosstalk between gut microbiota and pulmonary immunity, known as the gut-lung axis, where SCFAs serve as key mediators that modulate airway inflammatory responses and maintain homeostasis [22,23]. Among SCFAs, butyrate has attracted particular interest for its metabolic and anti-inflammatory properties. For example, butyrate enhances macrophage antimicrobial activity and anti-inflammatory activity by inhibiting histone deacetylases (HDACs) and activating the G protein-coupled receptors (GPRs), both of which influence cell metabolism [[24], [25], [26]]. Butyrate also sustains intestinal barrier integrity and exerts anti-inflammatory effects within the colon [27,28]. Nevertheless, the molecular mechanisms by which butyrate modulates immune responses in distal organs remain poorly understood.

In this study, we aimed to investigate the impact of butyrate on *K. pneumoniae -*induced pneumonia and elucidate the underlying mechanism. We demonstrate that butyrate mitigates *K. pneumoniae*-induced oxidative stress in alveolar macrophages (AMs) through activation of the p62-Keap1-Nrf2 axis, offering novel insights for the clinical management of bacterial pneumonia.

## 2 Materials and methods

### 2.1 Ethics statement

All experiments involving animals were conducted according to the Ethics Committee of Anhui Medical University (Approval no. LLSC20190253). All human studies were approved by the Ethics Committee for Clinical Research of the First Affiliated Hospital of Anhui Medical University (Approval no. PJ-2024-10-42). Written informed consent was obtained from all patients or their legal guardians.

### 2.2 Patients and clinical specimens

Peripheral blood samples were collected from 20 healthy donors and 39 patients diagnosed with pneumonia from the First Affiliated Hospital of Anhui Medical University. After centrifugation at 300*g* for 20 min at 4 °C, plasma was separated by centrifugation and stored at −80 °C, while the cellular fraction was processed for peripheral blood mononuclear cells (PBMCs) isolation using Lymphocyte Separation Medium (Solarbio, P8601). PBMCs were analyzed by flow cytometry, and plasma was used to measure the concentration of MDA. The clinical characteristics of bacterial pneumonia patients are shown in Table S4.

### 2.3 Animal

Wild-type C57BL/6 mice (8-10 weeks, 20-23 g) were purchased from GemPharmatech Co., Ltd. (Nanjin, Jiangsu). *p62*^*−/−*^, *Gpr43*^*−/−*^, *Gpr109a*^*−/−*^, *Nrf2*^*flox/flox*^ and *Lyz2*-Cre^+^ mice on a C57BL/6J background were purchased from Shanghai Model Organisms Center, Inc. Genotyping of knockout mice was performed using standard PCR protocols, and the primer sequences are presented in Table S1. All mice were housed under a specific pathogen-free environment at Anhui Medical University (Hefei, China). All experiments involving mice were approved by the Ethics Committee of Anhui Medical University. Experimental groups were matched for age and sex.

### 2.4 Bacterial strains

Carbapenem-resistant hypervirulent *K. pneumoniae* strain (CR-HvKP) was isolated from a patient in the First Affiliated Hospital of Anhui Medical University (Hefei, China). *Klebsiella pneumoniae* labeled with Green Fluorescent Protein (GFP-43816) was obtained from the Bacterial Resistance Surveillance Center of Anhui Province (Hefei, China). Bacteria were grown to logarithmic phase, frozen in Mueller-Hinton Broth (M-HB; OXOID, CM045B) containing 20% (v/v) glycerol, and stored at −80 °C. All bacterial strains were cultured in M − HB at 37 °C overnight before infection.

### 2.5 Mouse model of *K. pneumoniae* pneumonia and butyrate treatment

Mice were administered butyrate (150 mM) [26] in their drinking water for two weeks prior to infection. All mice were then anesthetized with pentobarbitalum natricum (10 mg/ml) and inoculated intranasally with 50 μL sterile phosphate-buffered saline (PBS) containing 1 × 10^5^ colony-forming units (CFU) of *K. pneumoniae*. Post-infection, mice were euthanized at various time points, and lung tissues were collected for analysis, including neutrophil infiltration, bacterial load, cytokines quantification, and histopathological assessment of lung injury.

### 2.6 Bronchoalveolar lavage fluid (BALF) collection and flow cytometry

BALF was collected at 6 and 48 h post-infection by instilling and retrieving 4 ml sterile PBS containing 0.5 mM EDTA using a 1.5 ml syringe. Retrieved cells were subjected to red blood cell lysis and incubated with anti-CD16/32 (Biolegend, 101320) to block the Fc, followed by staining with surface antibody with anti-CD45 (Biolegend, 368514), anti-CD11b (Biolegend, 550993), anti-CD11c (Biolegend, 128044), anti-Siglec-F (BD Pharmingen, 562681), anti-Ly-6G (BD Pharmingen, 56599), anti-Ly-6C (BD Pharmingen, 128044). After 30 min, cells were fixed using Cytofix/Cytoperm solution (BD Pharmingen, 554722) and permeabilized with Perm/Wash Buffer (BD Pharmingen, 554723). For intracellular oxidative stress detection, cells were stained with anti-4-HNE for 1 h and then washed thrice with sterile PBS. Finally, cells were analyzed using a FACS Celesta flow cytometer, and data were processed using FlowJo software.

### 2.7 Cell culture and treatment

Bone marrow-derived macrophages (BMDMs) and the mouse alveolar macrophage cell line MH-S were used in this study. Bone marrow cells were isolated from the femur and tibia of female mice by flushing the marrow using endotoxin-free Dulbecco's modified Eagle's medium (DMEM, Gibco, 8123489) supplemented with Penicillin-Streptomycin-Amphotericin B Solution (Procella, PB180120) through a sterile syringe. After centrifugation at 300*g* for 5 min at 4 °C, cells were collected and resuspended in DMEM containing 10% fetal bovine serum (v/v, Gibco, A5256701) and 20 ng/ml of M-CSF (Novoprotein, CB34). The cells were then cultured in a 37 °C incubator with 5% CO_2_ for 5-7 days to allow differentiation into BMDMs. MH-S cells (RRID: CVCL_3855) were purchased from Procell (Wuhan, China) and cultured in Roswell Park Memorial Institute 1640 (RPMI 1640, Gibco, 11875093) supplemented with 10% FBS and 1% Penicillin-Streptomycin-Amphotericin B Solution. For *in vitro* assays, cells were pretreated with butyrate (1 mM, Macklin, S817488) for 24 h, followed by infection with *K. pneumoniae* (multiplicity of infection [MOI] = 50). After 1 h of incubation, cells were washed thrice with sterile PBS and maintained in medium that was supplemented with gentamicin (100 ng/ml) until the subsequent experiment.

### 2.8 Vector construction and lentivirus production

The LentiCRISPRv2 plasmid was linearized by restriction digestion and subsequently ligated to annealed oligonucleotides (sequences provided in Table S3). To generate lentiviral particles, 293T cells were co-transfected with the lentiviral transfer vector alongside packaging plasmids ps. pAX2 and pMD. 2G. Viral supernatants harvested at 48- and 72-h post-transfection were concentrated and used for target cell transduction.

### 2.9 Protein extraction and western blot

Whole cells (from *in vitro* experiments) or lung tissues were homogenized in RIPA buffer (Beyotime Biotechnology, China) containing protease inhibitors cocktail and phosphatase inhibitors. The lysates were disrupted 2-3 times with an ultrasonic disruptor. Protein extracts were mixed with a loading buffer, denatured by boiling, and separated by SDS-PAGE. The separated proteins were transferred onto PVDF membranes and probed with primary antibodies. Detection was performed using enhanced chemiluminescence (ECL) substrate. The antibodies used were as follows: LC3B (L7543, Sigma-Aldrich, USA); p62 (23214), mTOR(2983), *p*-mTOR(5536) (all from Cell Signaling, USA), Nrf2 (A0674), Beclin1 (A7353), H2A.X (A11412), p-H2A.X (AP0687), GSTM2 (A13496), NQO1 (A23486), HO-1 (A1346), Bcl-2 (A19693)(all from ABclonal Biotechnology, China); Keap1 (10503-2-AP), Phospho-P62 (29503-1-AP), Caspase3 (19677-1-AP), BAX (50599-2-Ig), β-actin (66009-1-lg), GAPDH (10494-1-AP), AKT (10176-2-AP), *p*-AKT (66444-1-Ig)(all from Proteintech, China); 4-HNE (MA5-27570, Invitrogen, USA).

### 2.10 Immunofluorescence analysis

Cells were seeded onto confocal dishes and treated under the indicated conditions, followed by fixation with 4% paraformaldehyde for 15 min at room temperature. The cells were then permeabilized with 0.5% TritonX-100 for 30 min, blocked with 5% bovine serum albumin in PBST (0.5% Tween 20 in PBS) for 30 min, and incubated with the primary antibody at 4 °C overnight. After being washed three times with PBST, cells were incubated with the secondary fluorescent antibodies at 37 °C for 1 h. Finally, cells were stained with Hoechst 33258 (Sigma-Aldrich, 94403) which labeled the cell nucleus. All samples were determined with a Zeiss LSM-800 confocal microscope (Carl Zeiss, Germany).

### 2.11 Quantitative real-time PCR and RNA sequencing

Total RNA was extracted using TRIzol Reagent (Invitrogen, 15596018), and the reverse transcription was performed using PrimeScript RT Master Mix (Takara Bio, RR036A) under the following conditions: 37 °C for 15 min, 85 °C for 5 s. Gene expression was analyzed by quantitative real-time PCR (RT-qPCR) using TB Green *Premix Ex Taq* (Takara Bio, RR420A). The relative mRNA expression of each gene was normalized with respect to *β-actin* or *Gapdh* and reported as fold changes. The primers used are listed in Table S2. RNA-seq was performed as previously described [29]. The clean reads were aligned to the reference genome using Bowtie2 (version v2.2.5) and the gene expression values (fragments per kilobase per million mapped fragments, FPKM) of each sample were calculated by RSEM (version v1.2.8). Differentially expressed genes (DEGs) were detected as described in a previous study [30]. Pathways enriched with DEGs were annotated in the Kyoto Encyclopedia of Genes and Genomes (KEGG) and Gene Set Enrichment Analysis (GSEA). For GSEA, *p* < 0.05 and false discovery rate (FDR) *q*-value <0.25 were considered significantly enriched. Data from RNA sequencing of cells has been deposited in the NCBI database (PRJNA1301058, PRJNA1301807).

### 2.12 Histology examination and immunohistochemistry

Lung tissue was fixed in 4% paraformaldehyde (Biosharp, BL539A), dehydrated, embedded in paraffin and sectioned into 4 μm thickness. Hematoxylin and eosin (H&E) staining was performed according to the standard procedure. For immunohistochemistry, tissue sections were submerged into citrate antigen retrieval solution (Servicebio, G1202) to retrieve the antigens after deparaffinization and hydration. The slides were then submerged into 3% (v/v) H_2_O_2_ solution for 20 min to block endogenous peroxidase. After being washed three times with PBS, slides were blocked using 3% (v/v) Bovine Serum Albumin (BSA; Servicebio, GC305010) for 30 min and then incubated with primary antibody against MPO (1:500 dilution; Servicebio, GB11223) at 4 °C overnight. The next day, slides were washed with PBS and stained with horseradish peroxidase-conjugated Goat anti-Rabbit IgG (H + L; 1:200 dilution; Servicebio, GB23303) at room temperature for 1 h. Color development was performed using DAB (Solarbio, DA1016) followed by hematoxylin counterstaining. Slides were then dehydrated, cleared, mounted and scanned with a SLIDEVIEW VS200 Research Slide Scanner (Olympus, Japan). Images were analyzed using ImageJ software. At least five biological replicates were included in each group.

### 2.13 Elisa assay

Following euthanasia, lung tissues and serum samples were collected for inflammatory factors detection. Lung tissues were ground with sterile PBS, and serum was directly used for detection (1:10 dilution with sterile PBS). The concentrations of IL-1β, IL-6, and TNF-α were measured using ELISA kits (Dakewe Biotech Co. Ltd., China) according to the manufacturer's instructions. Absorbance was measured using a multifunctional microplate reader (Molecular Devices, Sunnyvale, CA, USA).

### 2.14 Apoptosis experiments *in vitro*

Cells were pretreated with butyrate (1 mM) for 12 h, followed by incubation with *K. pneumoniae* at various MOIs for 6 h. After centrifugation at 300*g* for 5 min, cells were collected and subjected to apoptosis detection using Annexin V-FITC Apoptosis Detection Kit (Beyotime Biotechnology, C1062L). After incubation, cells were detected by flow cytometry (FACS Celesta, BD Biosciences).

### 2.15 ROS measurement

Intracellular ROS levels were detected after co-culture with *K. pneumoniae* for 3 h using Reactive Oxygen Species Assay Kit (Beyotime Biotechnology, S0033S). Cells were incubated with DCFH-DA at 37 °C for 30 min. After being washed thrice with sterile PBS, the cells were collected and detected by flow cytometry.

### 2.16 Statistical analysis

Statistical analyzes were performed using GraphPad Prism 8.3.0 software. Data normality was assessed through Shapiro–Wilk test. Data were analyzed by two-tailed Student's *t*-test, Mann–Whitney *U* test, one-way ANOVA test or two-way ANOVA test. Data are presented as means ± standard deviation (SD). The Kaplan-Meier method was used to compare survival curves. The Pearson correlation analysis was performed to analyze the correlation between variables, and the index R representing Pearson's correlation coefficient. *p* < 0.05 was considered statistically significant.

## 3 Results

### 3.1 Butyrate mitigates *K. pneumoniae*-induced lung injury

**Fig. 1:**
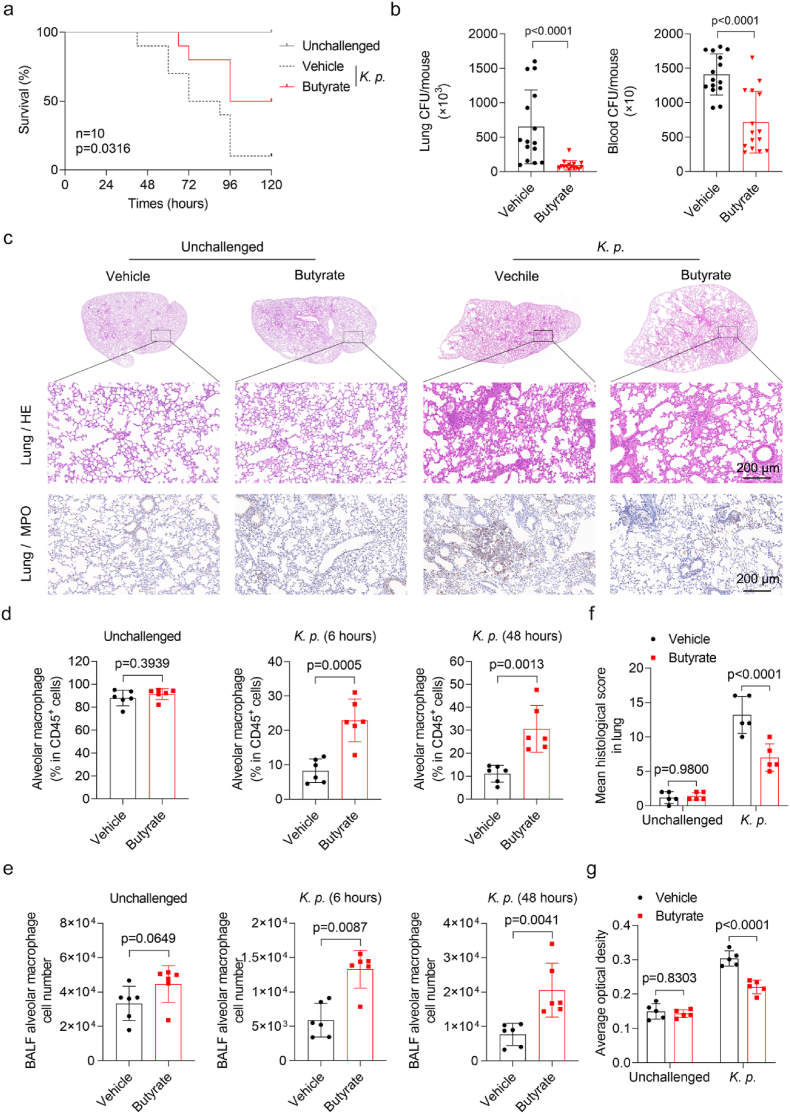
Butyrate attenuates *K. pneumoniae*-induced lung injury and rescues alveolar macrophages. (**a**) Survival rates of butyrate-treated mice and untreated mice infected with *K. pneumoniae* (n = 8 per group). (**b**) Pulmonary and blood bacterial burdens in butyrate-treated and untreated mice 24 h after infection with 1 × 10^5^ CFU of *K. pneumoniae* (n = 15 per group). (**c**) Representative images of H&E staining and MPO staining of lung sections (n = 5 per group). Scale bars, 200 μm. (**f-g**) Quantitative analysis of histological scores and MPO Immunohistochemical staining. (**d**) Percentage of alveolar macrophages among CD45^+^ cells in BALF from butyrate-treated and untreated mice at 6 and 48 h post-infection (n = 6 for per group). (**e**) Total numbers of alveolar macrophages in BALF (n = 6 per group). All data are presented as mean ± SD. *p* values were determined by two-tailed unpaired Student's *t*-test (b, d, e), two-way ANOVA (f, g), or log rank (Mantel-Cox) test (a).

To evaluate the effect of butyrate on *K. pneumoniae* infection, mice were pretreated with butyrate in drinking water for two weeks before the intratracheal challenge with *K. pneumoniae*, after which survival rate was determined. As shown in Fig. 1a, survival rate fell to 10% in untreated mice by 96 h, while 50% butyrate-treated mice survived. These findings suggest that butyrate improves survival in mice infected with *K. pneumoniae*. Consistent with this observation, butyrate supplementation significantly lowered bacterial loads in both lung tissue and blood (Fig. 1b). Pulmonary pathology was evaluated by H&E staining. Histological scoring revealed that butyrate treatment attenuated *K. pneumoniae*-induced lung injury, as evidenced by diminished inflammatory cell infiltration and preserved alveolar structure (Fig. 1c and f). Myeloperoxidase (MPO) activity in lung tissue is a specific marker of neutrophil influx leading to inflammation [31]. The immunohistochemistry (IHC) images (Fig. 1c) indicated markedly elevated MPO staining in infected lungs. Conversely, butyrate treatment significantly reduced MPO staining, indicating diminished tissue injury (Fig. 1c and g). Considering the crucial function exerted by the innate immune system on host defense, we then analyzed leukocyte populations in the bronchoalveolar lavage fluid (BALF) by flow cytometry. Notably, untreated infection caused a marked decrease in both the absolute number of alveolar macrophages and their percentage among CD45^+^ cells at 6 and 48 h post-infection (Fig. 1d and e). Mice treated with butyrate retained a greater number of alveolar macrophages and a higher fraction of CD45^+^ cells than controls (Fig. 1d and e, Fig. S1b). Butyrate also reduced both the number and the proportion of BALF neutrophils among CD45^+^ cells (Fig. S1c–d). Neither treatment affected the abundance of myeloid-derived monocytes (Fig. S1c–d). Collectively, these results demonstrate that butyrate treatment suppresses inflammation and mitigates *K. pneumoniae*-induced lung injury.

### 3.2 Butyrate promotes antioxidant process in alveolar macrophages under *K. pneumoniae* challenge

**Fig. 2:**
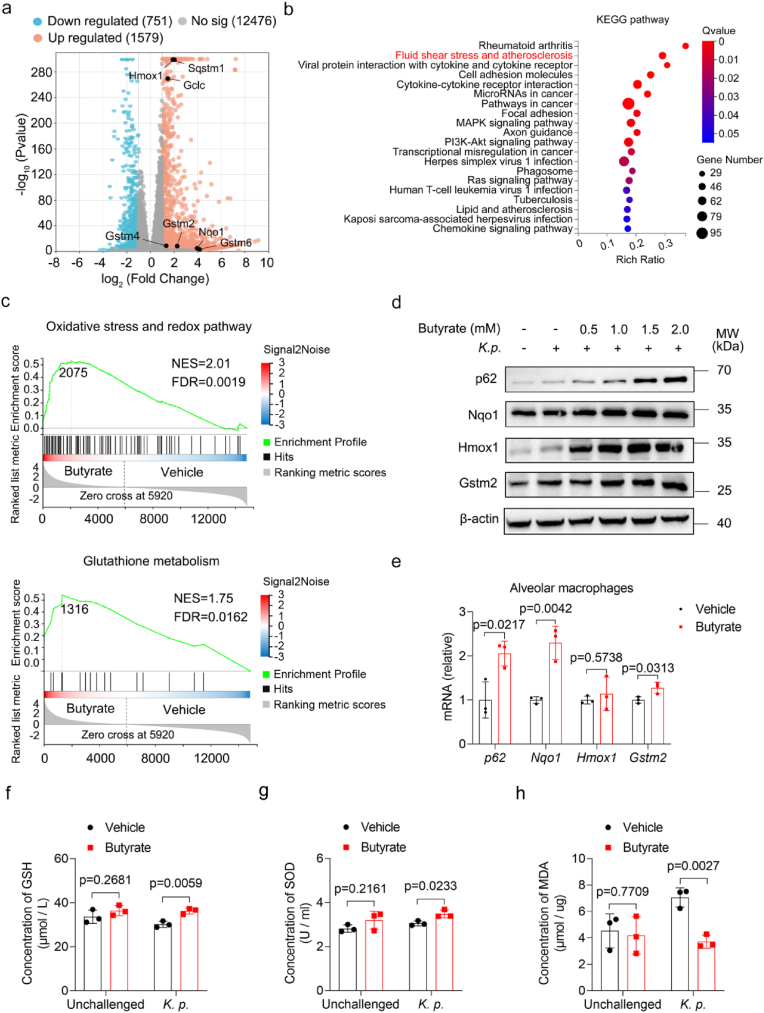
Butyrate promotes antioxidant process in alveolar macrophages under *K. pneumoniae* challenge. (a) Volcano plot of DEGs in MH-S cells from butyrate-treated (1 mM for 24 h) or untreated control, after infection with *K. pneumoniae* for 3 h (n = 4 per group). (b) KEGG pathways enrichment analysis of DEGs between butyrate-treated and untreated groups. The pathway related to antioxidant responses, is highlighted in red. (c) GSEA of selected DEGs. (d) Representative western blots of p62, Nqo1, Hmox1 and Gstm2 in MH-S cells treated with the indicated concentrations of butyrate for 24 h and then co-cultured with *K. pneumoniae* for 3 h. β-actin served as loading control. (e) RT-qPCR analysis of antioxidant gene expression in alveolar macrophages isolated from butyrate-treated and untreated mice (n = 3 per group). (f-h) Intracellular GSH, SOD and MDA concentrations in MH-S cells treated or untreated with butyrate after 3 h of *K. pneumoniae* infection (n = 3 per group). All data are presented as mean ± SD. *p* values were determined by two-tailed unpaired Student's *t*-test (e-h). NES, normalized enrichment score; FDR, false discovery rate. An FDR *q-*value <0.25 was considered significant.

Alveolar macrophages are the primary resident immune cells in the lung, which play a vital role in defending against pathogens and regulating inflammation to maintain pulmonary homeostasis [32,33]. Given our earlier observation that butyrate ameliorated *K. pneumoniae*-induced damage in alveolar macrophages, we performed RNA sequencing (RNA-seq) to investigate the transcriptional response of an alveolar macrophages cell line (MH-S) upon butyrate treatment. The Volcano plot revealed a total of 2330 differentially expressed genes (DEGs), comprising 751 downregulated genes and 1579 upregulated genes in the butyrate-treated group compared to controls (Fig. 2a). KEGG pathway enrichment analysis of these DEGs showed a significant activation of pathways associated with antioxidant signaling (Fig. 2b). A heatmap of representative DEGs showed marked upregulation of canonical antioxidant genes, including *Nqo1*, *Hmox-1*, *Gstms2*, *Gstms4, Gstms6, Gss* and *Gclm* (Fig. S2a and S2b). Interestingly, Sequestosome 1 (p62/Sqstm1), a known regulator that activates Nrf2, was also significantly upregulated [34]. Western blot validations confirmed increased expression of p62, Nqo1, Hmox-1 and Gstm2 proteins in butyrate-treated MH-S cells (Fig. 2d). Gene set enrichment analysis (GSEA) of RNA-seq data further validated that redox-related gene sets, including 'Oxidative stress and redox pathway' and 'Glutathione metabolism' were significantly enriched in butyrate-treated MH-S cells (Fig. 2c). Given that reduced glutathione (GSH) is a crucial antioxidant protecting cells from ROS [35], we observed significantly higher intracellular GSH levels in butyrate-treated MH-S cells compared to the untreated group (Fig. 2f). Consistently, intracellular superoxide dismutase (SOD) level was also elevated and malondialdehyde (MDA) level was decreased in the butyrate treatment group (Fig. 2g and h). To validate the RNA-seq results from MH-S *in vivo*, RT-qPCR was conducted on alveolar macrophages isolated from mice BALF. Consistently, butyrate treatment upregulated expression of *p62* and antioxidant genes including *Hmox1, Nqo1, Gstm2, Gss, Gclm in vivo* (Fig. 2e, Fig. S2c). To determine whether the antioxidant-promoting effect of butyrate is conserved in other macrophage populations, we performed RNA-seq on bone marrow-derived macrophages (BMDMs) from mice treated with butyrate. Similar to MH-S cells, redox-related gene sets were significantly upregulated in butyrate-treated BMDMs (Fig. S2e, Fig. S2g). KEGG enrichment also revealed activation of the p53 signaling and apoptosis pathways, which are associated with cellular stress responses and cell death (Fig. S2e). GSEA demonstrated that 'Oxidative Stress and Redox Pathway' and 'Glutathione Metabolism' were enriched in butyrate-treated BMDMs as well (Fig. S2d). Furthermore, we confirmed that butyrate treatment can also promote the expression of *p62, Hmox1, Nqo1* and *Gstm2* in BMDMs using RT-qPCR (Fig. S2f). Taken together, these results suggested that butyrate enhances antioxidant defense in macrophages by upregulating key redox-related pathways. This antioxidative response might contribute to the protective effect of butyrate against *K. pneumoniae-*induced oxidative stress and lung injury.

### 3.3 p62/SQSTM1 participants in the butyrate-mediated antioxidant process

**Fig. 3:**
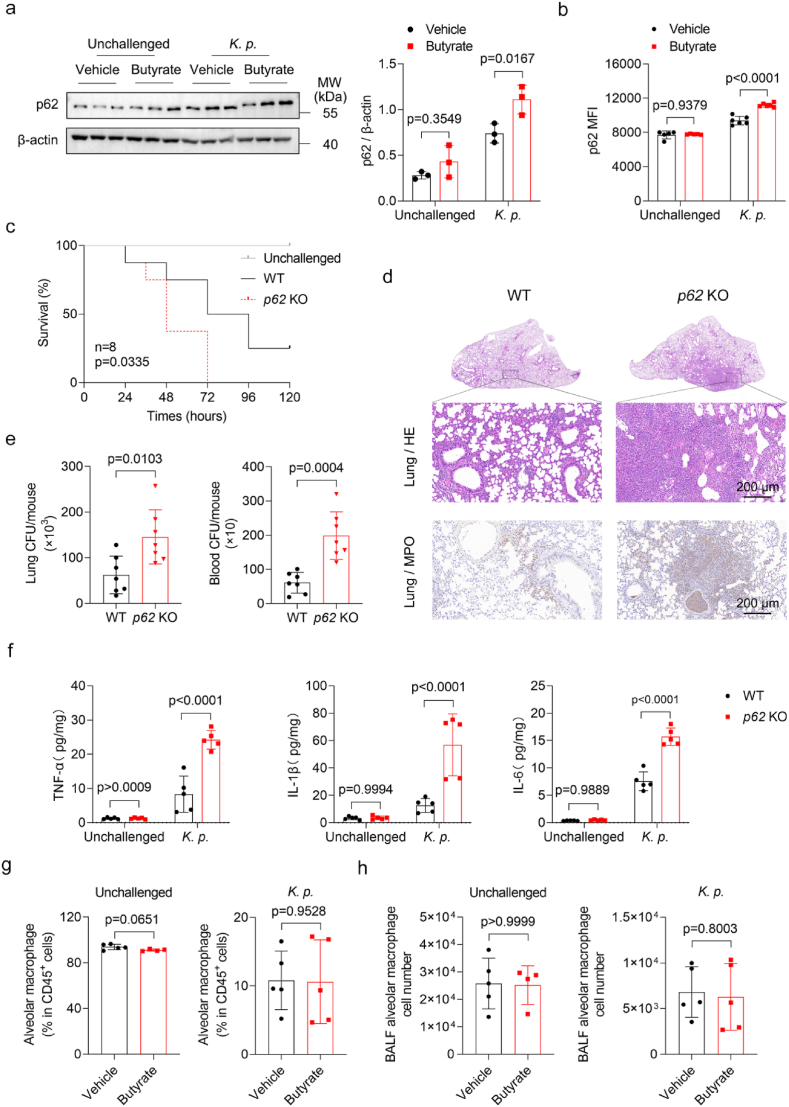
p62/SQSTM1 is essential for butyrate-mediated antioxidant process. (a) Representative Western blot analysis and densitometric quantification of p62 protein levels in lung tissues from butyrate-treated and untreated mice, and corresponding densitometric quantification (n = 3 per group). (b) Flow cytometry analysis of p62 expression in alveolar macrophages from butyrate-treated and untreated mice (n = 5 in control and butyrate group; n = 6 in *K. p.* and *K. p.* + Butyrate group). (c) Survival rates of *p62*^*+/+*^ or *p62*^*−/−*^ mice intranasally infected with *K. pneumoniae* (n = 8 per group). (d) Representative H&E staining and MPO staining images of lung sections from *p62*^*+/+*^ and *p62*^−/−^ mice (n = 5 per group). Scale bars, 200 μm. (e) Pulmonary and blood bacterial loads 24 h after *K. pneumoniae* infection (n = 7 per group). (f) The levels of proinflammatory cytokines TNF-α, IL-6, and IL-1β in lung tissues were detected by ELISA (n = 5 per group). (g-h) Percentage of alveolar macrophages among CD45^+^ cells and total alveolar macrophages counts in BALF from butyrate-treated and untreated *p62*^−/−^ mice at 6 h post-infection with *K. pneumoniae* (n = 5 per group). All data are presented as mean ± SD. *p* values were determined by two-tailed unpaired Student's *t*-test (e, g-h) and two-way ANOVA (a-b, f), or log rank (Mantel-Cox) test (c).

Previous studies showed that butyrate activates the Nrf2 antioxidant response in a p62-dependent manner in a mouse model of acute liver injury [36]. However, it is unclear whether butyrate mediates macrophage antioxidant response via p62 in *K. pneumoniae*-induced pneumonia. Notably, our data show that butyrate treatment increases p62 expression in MH-S macrophages *in vitro* (Fig. 2d). We then confirmed this result through immunofluorescence staining (Fig. S3f). To understand the effect of *K. pneumoniae* on p62 expression, we performed Western blot analysis of MH-S infected at varying multiplicities of infection (MOI). The results showed that p62 protein levels significantly increased at MOI was greater than 20 (Fig. S3e). We next validated this finding in BMDMs and the p62 protein levels were increased at an MOI of 10 (Fig. S3e). Furthermore, butyrate induced *p6*2 mRNA in a time- and dose-dependent manner during *K. pneumoniae* infection*,* with maximal activation at 1 mM for 12 h (Fig. S3c–d). To validate this *in vivo*, we measured p62 protein levels in lung tissue and alveolar macrophages of infected mice. Compared with controls, butyrate-treated mice exhibited elevated p62 protein levels in both lung tissue and alveolar macrophages (Fig. 3a and b). These data, together with RNA-seq findings, provide preliminary evidence that p62 might mediate butyrate's antioxidant effects in macrophages. To test this hypothesis, we established a pneumonia model in *p62*-knockout (*p62*^*−/−*^) mice (Fig. S10a). After *K. pneumoniae* infection, *p62*^*−/−*^ mice exhibited a higher mortality than wild-type group (Fig. 3c), correlating with elevated pro-inflammatory cytokines TNF-α, IL-6 and IL-1β in the lung tissue and TNF-α, IL-6 in the serum (Fig. 3f, Fig. S3i), and elevated bacterial loads in lung and blood (Fig. 3e). Consistent with the *in vivo* results, we measured the expression of pro-inflammatory cytokines in BMDMs using RT-qPCR and found that *Tnfα*, *Il6*, *Il1β*, *Il12a*, and *Il12b* were upregulated in *p62*-deficient macrophages (Fig. S3j). Histopathological analysis revealed pronounced inflammatory infiltration and alveolar destruction in *p62*^*−/−*^ mice (Fig. 3d, Fig. S3a). IHC confirmed increased neutrophil infiltration in *p62*^*−/−*^ lungs (Fig. 3d, Fig. S3b). Furthermore, p62 deletion abolished butyrate's effects on alveolar macrophages, monocytes, and neutrophils in BALF (Fig. 3g–h, Fig. S3g–h).

### 3.4 Butyrate mediates antioxidant response via the p62–Keap1–Nrf2 axis

**Fig. 4:**
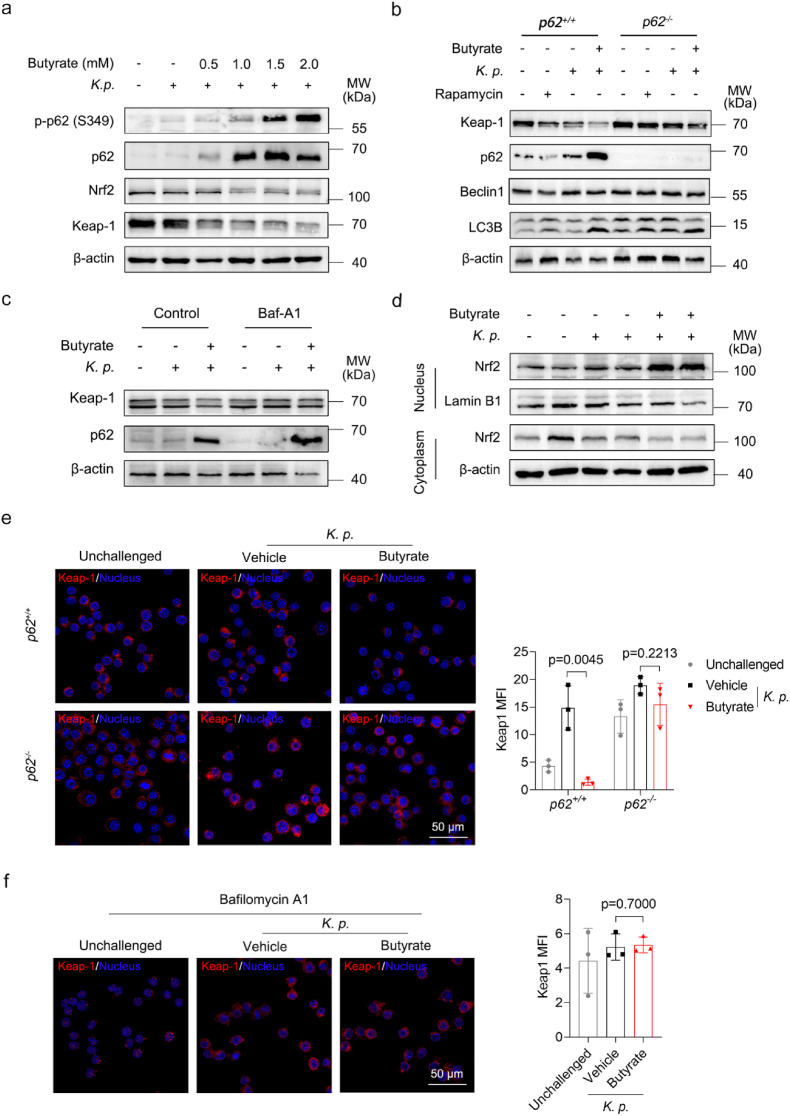
Butyrate mediates the antioxidant response via p62–Keap1–NRF2 axis. (a) Representative Western blot showing p-p62 (S349), p62, Nrf2 and Keap1 in MH-S cells challenged with *K. pneumoniae* and treated with the indicated concentrations of butyrate. (b) Representative Western blot of Keap1, p62, Beclin1 and LC3B in MH-S cells pretreated with butyrate (1 mM, 24 h) or rapamycin (100 μM, 1 h) prior to *K. pneumoniae* infection. (c) Representative Western blot of Keap1 and p62 in Bafilomycin A1-treated (100 nM for 2 h) and untreated MH-S cells. (d) Representative Western blot of Nrf2 in cytoplasm and nucleus in butyrate-treated (1 mM, 24h) and untreated MH-S cells. (e) Representative immunofluorescence images and quantification analysis of the mean fluorescence intensity of Keap1 in butyrate-treated and untreated *p62*^*+/+*^ or *p62*^*−/−*^ MH-S cells following *K. pneumoniae* infection (n = 3 per group). The nuclei were stained with Hoechst33258 (blue), and *K. pneumoniae* was stained with CFSE. Scale bars, 50 μm. (f) Representative immunofluorescence images and quantification analysis of the mean fluorescence intensity of Keap1 in Bafilomycin A1-treated MH-S cells (n = 3 for each group). Scale bars, 50 μm. All data are presented as mean ± SD. *p* values were determined by two-tailed unpaired Student's *t*-test (e-f).

The Keap-Nrf2 pathway regulates antioxidant enzyme production and cellular redox homeostasis [7]. Our findings suggest that butyrate modulates Nrf2 signaling by inducing p62. To test this hypothesis, we treated MH-S cells with increasing concentrations of butyrate. Cytosolic levels of Nrf2 and Keap1 decreased gradually in a dose-dependent manner after butyrate treatment, whereas p62 was upregulated in a butyrate dose-dependent manner (Fig. 4a). Butyrate exerts downstream signaling via the membrane receptors Gpr43/Gpr109a and HDAC inhibition [37]. To investigate the mechanism underlying butyrate-induced p62 upregulation, we obtained and bred *Gpr43*-knockout and *Gpr109a*-knockout mice (Fig. S10c–d). Using BMDMs from *Gpr43*^*−/−*^ and *Gpr109a*^*−/−*^ mice, we verified *in vitro* that butyrate enhances p62 expression in a Gpr43-dependent manner, but does not alter *Hdac1* or *Hdac3* expression in WT or *Gpr43*^*−/−*^ BMDMs (Fig. S5a–c). Under basal conditions, Nrf2 is bound to Keap1 and degraded via the ubiquitin-proteasome pathway [38]. Accumulation of p62 competitively binds Keap1 and prevents Nrf2 degradation [16]. Phosphorylation of p62 enhances its affinity for Keap1 [17]. Our data showed that butyrate induced p62 phosphorylation and dose-dependently increased phosphorylated p62 accumulation (Fig. 4a). Of note, we found that the PI3K-AKT-mTOR signaling pathway in macrophages was upregulated after butyrate treatment (Fig. S5d). Since mTOR has been reported to be a critical kinase that promotes p62 phosphorylation [17], we examined the protein expression of the related pathway by Western blotting. The results showed that butyrate promoted the phosphorylation of AKT and mTOR in a dose-dependent manner (Fig. S5e). In addition, we found that butyrate treatment had no effect on the expression of *Tbk1* and *Csnk2a2* in macrophages, which are two other critical kinases that promote p62 phosphorylation [39,40] (Fig. S5f). p62 acts as a cargo receptor for autophagic degradation of ubiquitinated target, which mediates Keap1 degradation via autophagy [14,34]. Considering autophagy's role in immunity [41], we evaluated whether butyrate modulates autophagy in *K. pneumoniae*-infected macrophages. As expected, LC3B protein levels increased following butyrate treatment regardless of p62 status, indicating enhanced autophagic flux (Fig. 4b, Fig. S4a). Consistent with protein data, the mRNA expression level of *LC3* was also elevated by butyrate, while there was no significant difference in *Becn1* expression (Fig. S4d). Western blot and immunofluorescence revealed that butyrate decreased Keap1 protein levels in wild-type cells, but not in p62-deficient macrophages (Fig. 4b and e). Western blot results of lung tissues further confirmed that p62 deficiency leads to abnormal degradation of Keap1 (Fig. S4c). However, butyrate had no effect on *Keap1* mRNA in either *p62*^*+/+*^ or *p62*^*−/−*^ MH-S cells (Fig. S4e). To confirm autophagy involvement, we treated cells with bafilomycin A1 to block autophagosome-lysosome fusion. As a consequence, Keap1 accumulated in the cytoplasm irrespective of butyrate treatment (Fig. 4c and f). Nrf2 mediates cellular antioxidant responses by binding antioxidant response elements (AREs) within gene promoters [42]. We then separated cytosolic and nuclear fractions to assess Nrf2 localization. The results showed that butyrate reduced cytosolic Nrf2 and Keap1 levels while enriching Nrf2 in the nuclear fraction (Fig. 4d, Fig. S4b). Moreover, butyrate failed to induce Nrf2 and its target antioxidants (*Hmox1, Nqo1)* in *p62*^*−/−*^ MH-S cells (Fig. S4f–g). In line with previous reports [34], our results reveal a positive feedback loop in which butyrate-induced p62 expression activates Nrf2 and its target genes, in turn, upregulating p62 (Fig. S3c–d). In summary, these data support that butyrate promotes p62-dependent autophagic degradation of Keap1 and nuclear translocation of Nrf2 in macrophages during *K. pneumoniae* infection. We establish butyrate as a key mediator which links autophagy and oxidative stress.

### 3.5 Conditional disruption of Nrf2 in macrophages attenuates butyrate-mediated protection against *K. pneumoniae* infection

**Fig. 5:**
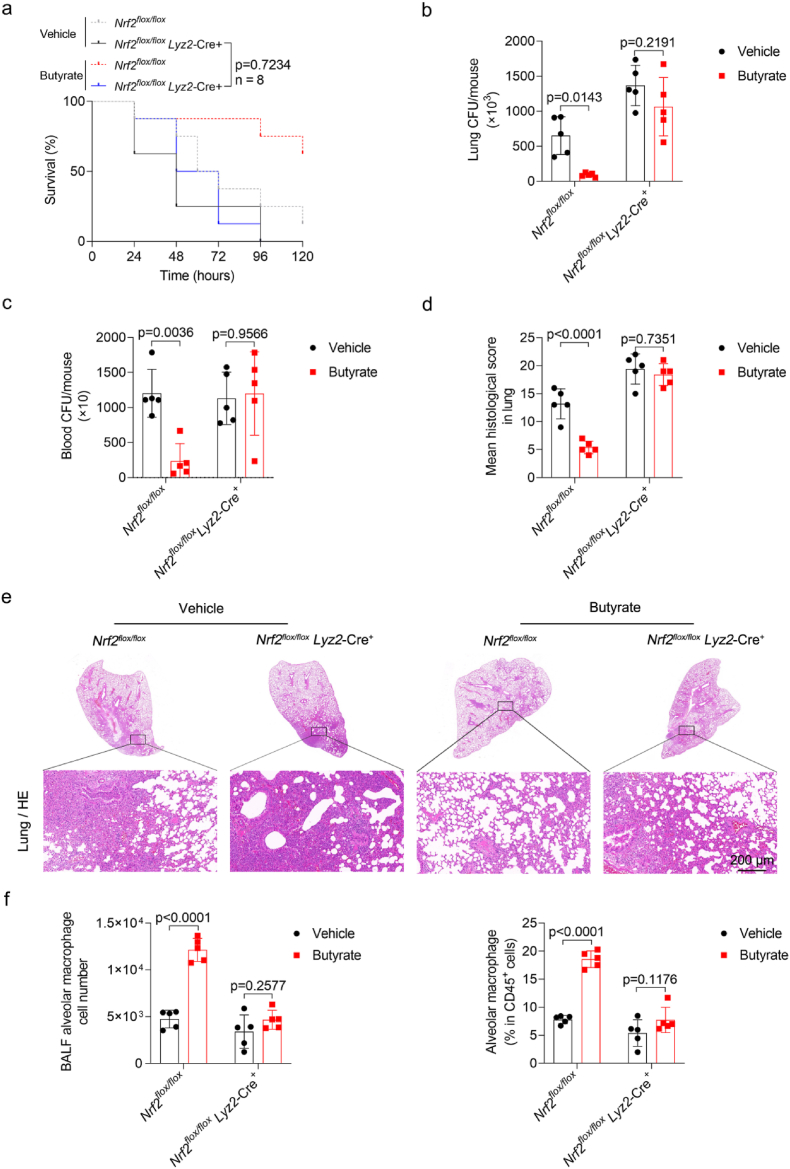
**Conditional disruption of Nrf2 in macrophages attenuates butyrate-mediated protection against *K. pneumoniae* infection** (a) Survival of *K. pneumoniae*-infected *Nrf2*^*flox/flox*^ and *Nrf2*^*flox/flox*^*Lyz2*-Cre^+^ mice with or without butyrate treatment (n = 8 per group). (b-c) Pulmonary and blood bacterial burdens in *K. pneumoniae*-infected *Nrf2*^*flox/flox*^ and *Nrf2*^*flox/flox*^*Lyz2*-Cre^+^ mice with or without butyrate treatment (n = 5 per group). (d) Quantitative analysis of histological scores in each group (n = 5 per group). (e) Representative images of H&E staining of lung sections (n = 5 per group). Scale bars, 200 μm. (f) Total numbers of alveolar macrophages and percentage of alveolar macrophages among CD45^+^ cells in BALF from *Nrf2*^*flox/flox*^ and *Nrf2*^*flox/flox*^*Lyz2*-Cre^+^ mice with or without butyrate treatment (n = 5 per group). All data are presented as mean ± SD. *p* values were determined by two-way ANOVA (b, c, d, f), or log rank (Mantel-Cox) test (a).

To investigate whether the functions of Nrf2 on macrophages are relevant to butyrate-mediated protection during *K. pneumoniae* infection, we purchased Nrf2-floxed gene-edited mice and crossed them with *Lyz2*-Cre ^+^ mice to generate *Nrf2*^*flox/flox*^ *Lyz2*-Cre^+^ mice (Fig. S10b). We observed that butyrate-treated *Nrf2*^*flox/flox*^ *Lyz2*-Cre ^+^ mice had a similar mortality after *K*. *pneumoniae* infection compared to untreated *Nrf2*^*flox/flox*^ *Lyz2*-Cre^+^ mice (Fig. 5a). In line with observations *in vitro*, bacterial burdens in the lungs and blood of these two groups showed no significant difference, while *Nrf2*^*flox/flox*^ mice under butyrate treatment showed lower bacterial burdens (Fig. 5b and c). Consequently, butyrate alleviates mouse lung tissue pathological injury in a Nrf2-dependent manner (Fig. 5d and e). Moreover, the number and proportion of alveolar macrophages in BALF were significantly increased in butyrate-treated *Nrf2*^*flox/flox*^ mice compared with the untreated group, whereas no significant statistical difference was found in butyrate-treated *Nrf2*^*flox/flox*^ *Lyz2*-Cre^+^ mice (Fig. 5f, Fig. S6a). In addition, we observed that butyrate promoted the expression of GSH and SOD in lung tissue and reduced the content of MDA in a Nrf2-dependent manner (Fig. S6b). Accordingly, these results confirm that butyrate alleviates *K. pneumoniae*-induced lung injury in a macrophage Nrf2-dependent manner.

### 3.6 Butyrate alleviates *K. pneumoniae*-induced oxidative stress both *in vivo* and in vitro

**Fig. 6:**
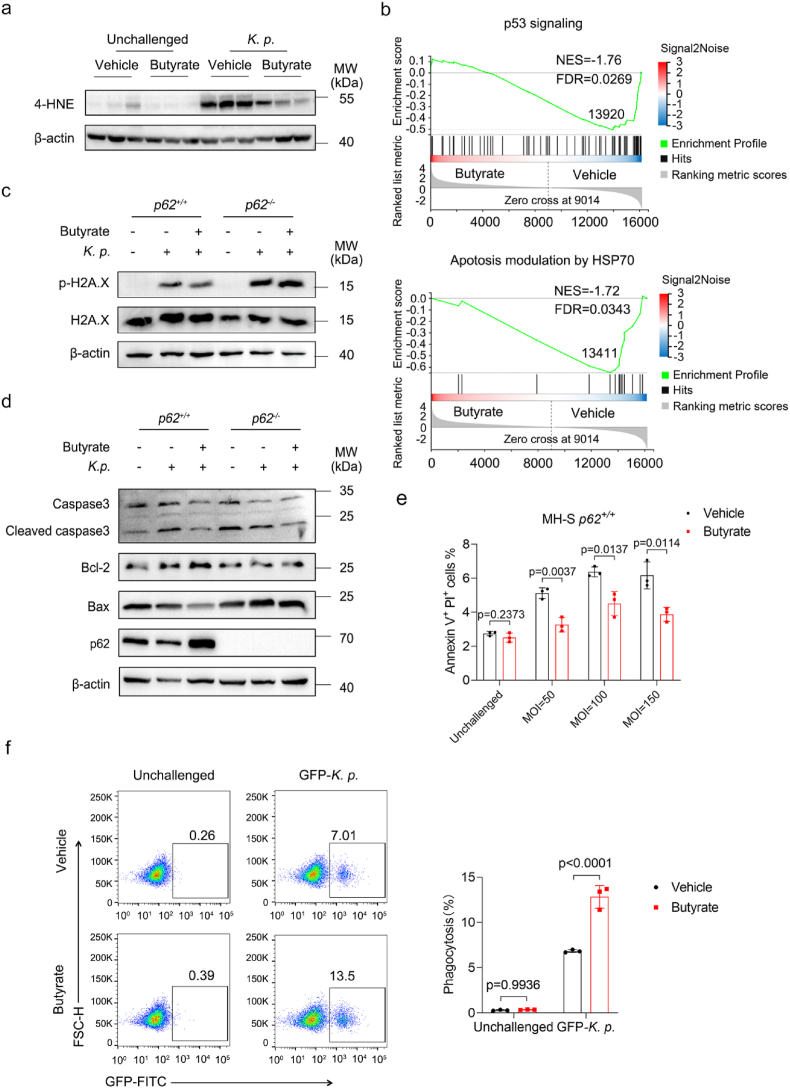
Butyrate alleviates *K. pneumoniae-*induced oxidative stress both *in vivo* and *in vitro*. (a) Representative Western blot of 4-HNE in lung tissues from butyrate-treated and untreated mice (n = 3 per group). (b) GSEA of indicated DEGs in butyrate-treated versus untreated macrophages under *K. pneumoniae* infection. (c) Representative Western blot of H2AX and p-H2AX in *p62*^*+/+*^ or *p62*^*−/−*^ MH-S cells treated with or without butyrate and infected with *K. pneumoniae* for 3 h. (d) Representative Western blot of p62, Caspase3, Cleaved caspase3, Bcl2 and Bax in *p62*^*+/+*^ or *p62*^*−/−*^ MH-S cells treated with or without butyrate and infected with *K. pneumoniae* for 3 h. (e) Percentage of Annexin V^+^/PI^+^ cells in butyrate-treated and untreated MH-S cells infected with the indicated MOI of *K. pneumoniae* for 6 h (n = 3 per group). (f) Quantification analysis of the phagocytosis rate in butyrate-treated and untreated *p62*^*+/+*^ or *p62*^*−/−*^ MH-S cells infected with GFP-*K. pneumoniae* for 30 min (n = 3 for each group). All data are presented as mean ± SD. *p* values were determined using a two-tailed unpaired Student's t-test (e) and two-way ANOVA (f).

Since butyrate promotes antioxidant defenses in alveolar macrophages, we next established a *K. pneumoniae* pneumonia mouse model to determine whether butyrate reduces *K. pneumoniae*-induced oxidative stress *in vivo*. Western blot analysis of 4-HNE in lung tissues suggested butyrate reduced *K. pneumoniae*-induced 4-HNE accumulation (Fig. 6a, Fig. S7a). Consistent with this result, the MDA levels in lung tissue were significantly lower in the butyrate-treated group, while the levels of SOD and GSH were increased (Fig. S7b–d). Next, we evaluated oxidative damage in MH-S cells infected with *K. pneumoniae in vitro*. GSEA of RNA-seq data revealed that apoptosis-related gene sets were upregulated in the vehicle group compared to the butyrate-treated group, including 'p53 signaling' and 'Apotosis modulation by HSP70', which have been reported to be involved in regulating cell cycle arrest, apoptosis and DNA repair [43](Fig. 6b). We next performed Western blot analysis of H2A.X and phosphorylated histone H2A.X (p-H2A.X, a marker of DNA damage) [44] in infected MH-S cells, and the p-H2A.X protein level was significantly attenuated by butyrate in a p62-dependent manner (Fig. 6c). In addition to DNA damage, oxidative stress can trigger apoptosis [44]. As shown in the heatmap analysis, the expression of Caspase 3 was decreased while the expression of Bcl2 was increased in the butyrate-treated group (Fig. S7g). The next Western blotting determined that protein levels of cleaved caspase-3 (C-caspase3) and Bax were increased after *K. pneumoniae* infection, whereas butyrate reversed these changes and upregulated Bcl-2 (Fig. 6d). Moreover, *p62* deficiency enhanced apoptosis. To further validate these results, we co-cultured MH-S cells with *K. pneumoniae* at varying MOI and measured apoptosis by flow cytometry. Similarly, *K. pneumoniae* infection induced macrophages apoptosis in a MOI-dependent manner, while butyrate reduced apoptotic proportions (Fig. 6e, Fig. S7e). *p62* deficiency further promoted macrophages apoptosis under *K. pneumoniae* infection (Fig. S7f). Besides, Nacetylcysteine (NAC), a pharmacological antioxidant, could reduce apoptotic proportions of macrophages in both wild-type and *p62*-deficient cells (Fig. S7h). To further investigate whether oxidative stress affects macrophage phagocytosis and bactericidal activity, we co-cultured MH-S cells with GFP-labeled *K. pneumoniae* and evaluated phagocytosis using flow cytometry. As shown in Fig. 6f, butyrate enhanced macrophage phagocytosis at 30 min post-infection. While phagocytic ROS generation is essential for microbicidal activity, we examined intracellular ROS both in wild-type and *p62*-deficient MH-S cells. Subsequent data confirmed that the *K. pneumoniae* infection increased ROS production in MH-S cells and that p62 deficiency further elevated ROS accumulation (Fig. S7i). Both *in vitro* and *in vivo* data suggest that butyrate might alleviate *K. pneumoniae*-induced oxidative stress.

### 3.7 Increased oxidative stress in *K. pneumoniae* pneumonia patients

**Fig. 7:**
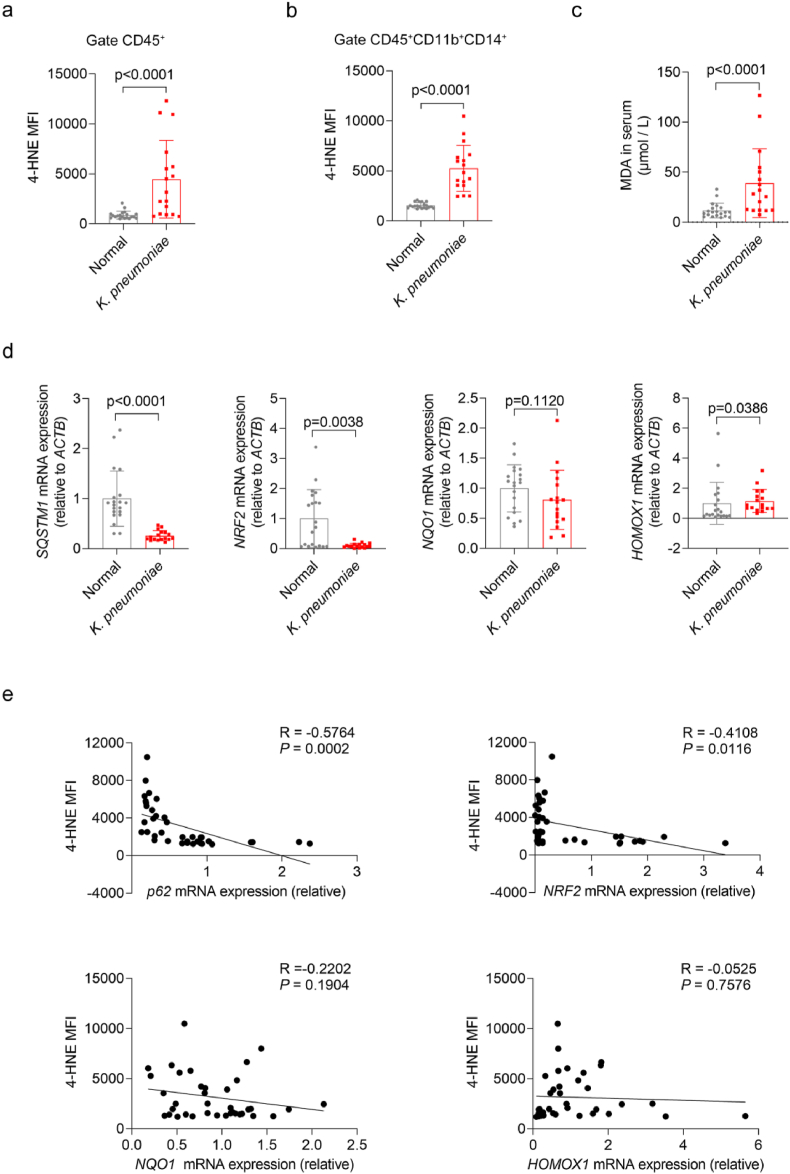
**Increased oxidative stress in *K. pneumoniae* pneumoniae patients** (a-b) Flow cytometric analysis of 4-HNE mean fluorescence in CD45^+^ cells and CD11B^+^CD14^+^ monocytes from normal individuals (n = 20) and patients with *K. pneumoniae* pneumonia (n = 17). (c) Concentration of MDA in serum from normal individuals (n = 20) and patients with *K. pneumoniae* pneumonia(n = 17). (d) RT-qPCR analysis of *SQSTM1*, *NRF2, NQO-1 and HO-*1 mRNA expression in PBMCs from normal individuals (n = 20) and patients with *K. pneumoniae* pneumonia(n = 17). (e) Linear regression analysis of *SQSTM1*, *NRF2, NQO-1 and HO-*1 mRNA expression with 4-HNE production in PBMCs (normal individuals, n = 20; patients, n = 17). All data are presented as mean ± SD. *p* values were determined using two-tailed unpaired Student's t-test (a-d).

Giving the findings above, we demonstrated that *K. pneumoniae* infection induces oxidative stress in macrophages both *in vivo* and *in vitro*, with p62, Nrf2 and its targeted antioxidant genes contributing to butyrate-mediated antioxidant responses. However, whether similar oxidative stress injury occurs in clinical patients with *K. pneumoniae* pulmonary infection remains unclear. To address this, we collected peripheral blood samples from patients with bacterial pneumonia and assessed oxidative damage markers in both peripheral blood mononuclear cells (PBMCs) and plasma. Patients were stratified into two groups based on the causative bacterial pathogen: *K. pneumoniae* and non-*K. pneumoniae* bacterial pneumonia groups. Plasma malondialdehyde (MDA) was measured. Compared to healthy controls, plasma MDA levels were elevated in *K. pneumoniae* pneumonia groups, while there were no significant differences in non-*K. pneumoniae* bacterial pneumonia groups (Fig. 7c, Fig. S8f). However, both patient groups exhibited an accumulation of 4-hydroxy-2-nonenal (4-HNE) both in CD45^+^ cells and peripheral circulating monocytes (Fig. 7a and b Fig. S8d-e). 4-HNE is a major lipid-peroxidation product that causes protein dysfunction and nucleic acid damage [45,46]. We then examined the mRNA expression levels of *p62*, *NRF2*, *Nqo1* and *HO-1* in PBMCs from all groups. The mRNA levels of *p62* and *NRF2* were significantly lower in pneumonia patients compared with controls (Fig. 7d, Fig. S8g). In addition, the levels of 4-HNE in circulating monocytes negatively correlated with the expression of *p62* and *NRF2* but not with *Nqo1* or *HO-1* in *K. pneumoniae* pneumonia groups (Fig. 7e). These results suggest that butyrate-dependent p62 signaling is essential for cellular antioxidant defense during *K. pneumoniae* infection. To investigate whether butyrate can alleviate oxidative damage in patients with *K. pneumoniae* infection, we isolated PBMCs from the patients and co-incubated them with butyrate *in vitro*. The level of 4-HNE was then detected by flow cytometry, and the results demonstrated that butyrate reduced the 4-HNE level in CD45^+^ cells and monocytes *in vitro* (Fig. S9a). In addition, butyrate also upregulated the expression of *p62* and antioxidant stress-related genes in PBMCs (Fig. S9b). Collectively, these findings indicate that patients with pulmonary infections caused by *K. pneumoniae* or other bacterial pathogens exhibit elevated oxidative stress in circulating monocytes, and butyrate exhibits potential therapeutic value.

## 4 Discussion

Macrophages detect invading pathogens via pattern-recognition receptors and rapidly neutralize them through phagocytosis [47,48]. This phagocytic process triggers robust ROS production—essential for intracellular bacterial killing—primarily generated through mitochondrial respiration and NADPH oxidase (NOX2) activity on phagosomal membranes [5,49]. Crucially, cellular homeostasis depends on a delicate balance between ROS generation and elimination. When this equilibrium is disrupted, excessive ROS accumulation induces lipid peroxidation, culminating in oxidative cellular damage [50]. 4-HNE, a reactive aldehyde generated during lipid peroxidation, is a key mediator of oxidative tissue damage, which has been demonstrated to exacerbate acute lung injury by impairing mitochondrial function [51]. Moreover, 4-HNE exerts its cytotoxic effects through multiple mechanisms, including protein carbonylation and nucleic acid adduct formation [45,46]. Despite its established role in acute lung injury and chronic obstructive pulmonary disease [52], the contribution of 4-HNE to pathogen-driven conditions such as bacterial pneumonia remains poorly characterized. In the present study, we provide evidence that pulmonary infection with *K. pneumoniae* induces oxidative stress in murine lung tissues, as indicated by the significantly increased accumulation of 4-HNE and MDA. This observation is further supported by findings from clinical specimens: analysis of PBMCs isolated from patients with bacterial pneumonia demonstrated elevated levels of 4-HNE adducts. Notably, such elevation was not restricted to *K. pneumoniae*-associated infections but was also observed in cases caused by other common bacterial pathogens, including *Pseudomonas aeruginosa*, *Acinetobacter baumannii*, and *Staphylococcus aureus*. Further investigation of murine BALF showed that *K. pneumoniae* infection triggered a pronounced reduction of AMs. Given that oxidative stress is a known inducer of macrophage death [44,53], our results confirmed that *K. pneumoniae* infection induced DNA damage and apoptosis in AMs. Collectively, these observations suggest that *K. pneumoniae* infection induces oxidative stress in monocytes/macrophages, which may be a critical driver of the depletion of AMs.

The connection between gut microbiota and lung immunity is well established [23,54]. SCFAs, the most abundant microbiota metabolites, exert immunomodulatory functions and serve as host energy substrates [55]. Our previous study highlighted that gut microbiota protect against bacterial-induced pneumonia and that SCFAs promote macrophage phagocytosis of *K. pneumoniae* [29,56]. Consistent with our results, the SCFA butyrate shows potent activity against Gram-negative bacteria by inhibiting HDACs [26]. In addition to antimicrobial activity, SCFAs exhibit anti-inflammatory properties via G protein-coupled receptors (GPRs), especially in the intestines [25,57]. However, the mechanism underlying SCFAs' immunomodulatory functions in pulmonary bacterial infection remains unclear. Our findings extend previous studies, showing that butyrate is crucial for maintaining redox balance in AMs. Butyrate mitigates *K. pneumoniae-*induced pneumonia by activating a p62-dependent antioxidant pathway and preserving alveolar macrophages. In the absence of p62, Nrf2 nucleus translocation and its downstream gene expression were diminished. AMs are a subset of tissue-resident macrophages localized in the alveolar space, which defend against pathogens and regulate lung inflammation [33]. Respiratory infections with bacteria and viruses have been demonstrated to cause severe depletion of fetal monocyte-derived AMs, while maintaining adequate AMs is crucial for proper antibacterial host defense [53,58,59]. Consistent with previous research, acute *K. pneumoniae* lung infection reduced AMs numbers and increased pulmonary neutrophils, whereas butyrate restored AMs and limited neutrophil recruitment. Transcriptomic analysis revealed that butyrate activates antioxidant pathways in alveolar macrophages. We further demonstrated that butyrate treatment reduced *K. pneumoniae-*induced oxidative damage in AMs and promoted the production of antioxidant enzymes, such as GSH and SOD. Moreover, butyrate-treated macrophages exhibit enhanced phagocytic and bactericidal capacities, and this result is consistent with findings from the murine pneumonia model. Our findings indicate that butyrate exerts antioxidant protection and protects macrophages against *K. pneumoniae-*induced oxidative stress.

Mechanistically, we demonstrate that butyrate upregulates p62 expression, activates NRF2, and subsequently initiates a downstream antioxidant transcriptional program. The signaling adaptor p62 harbors multiple functional domains, among which the Keap1-interacting region (KIR) binds to Keap1 at the Nrf2-binding pocket, thereby stabilizing Nrf2 by preventing its degradation [16]. Phosphorylation of p62 augments its binding affinity for Keap1, which in turn promotes Nrf2 activation [17]. Our data further reveal that butyrate upregulates the p62-Keap1-Nrf2 axis and facilitates Nrf2 nuclear translocation under *Klebsiella pneumoniae* challenge. Notably, we observed concentration-dependent phosphorylation of p62, indicative of butyrate-mediated regulation of p62 post-translational modification. Consistent with these results, butyrate promotes AKT and mTOR phosphorylation in a concentration-dependent manner. Given that mTOR acts as a pivotal kinase responsible for p62 phosphorylation, our findings suggest that butyrate may participate in this process by activating the PI3K-AKT-mTOR signaling cascade. Nrf2 activation induces a battery of antioxidative genes that eliminate cellular ROS and reduce oxidative damage. Nrf2 also regulates p62 expression by binding a conserved ARE directly in its promoter/enhancer, forming a positive feedback loop [34]. This feedback likely explains the time-dependent rise in p62 after butyrate treatment. Moreover, our results demonstrate that knockout of the butyrate receptor *Gpr43* abolishes this stimulatory effect, confirming that Gpr43 acts as a key upstream gene in the regulation of *p62* expression by butyrate. In addition to redox control, Nrf2 acts as an anti-inflammatory transcription factor in macrophages by suppressing pro-inflammatory cytokine transcription [9]. Therefore, we propose that butyrate may regulate inflammatory responses in pulmonary infections through the p62-Nrf2 axis. Indeed, multiple studies have reported that butyrate participates in the anti-inflammatory response [28,60,61]. In murine pneumonia models, our results revealed butyrate treatment inhibited neutrophil infiltration and attenuated pulmonary inflammation during *K. pneumoniae* infection; conversely, these protective effects were abolished in *p62*^*−/−*^ mice. Following *p62* deletion, macrophages exhibited elevated expression of pro-inflammatory cytokines in response to infection. Whether butyrate's anti-inflammatory effect is mediated by the p62-Nrf2 signaling pathway remains to be clarified.

Under basal conditions, Nrf2 is bound by Keap1 and targeted for degradation through the ubiquitin-proteasome pathway. Keap1 serves as an adaptor of a Cul3-containing E3 ubiquitin ligase that ubiquitinates Nrf2 [10]. Factors such as p62 [16], Nestin [62] and iASPP [63] interact with the Nrf2-Keap1 complex, inhibit Keap1's activity, and thereby activate Nrf2 signaling. Unlike Nrf2, inactivated Keap1 is degraded via autophagy [64]. Autophagy is a conserved intracellular digestion and recycling system involving the delivery of cytoplasmic cargo to the lysosome, and emerging evidence suggests that it also maintains redox homeostasis [65,66]. Consistent with these findings, we demonstrated that butyrate stabilized Nrf2 by promoting autophagic degradation of Keap1 in macrophages. This process was mediated by p62, which serves as a selective autophagy receptor that binds to LC3 and targets ubiquitinated proteins for degradation (including Keap1) [14,34]. In this study, we observed that *K. pneumoniae* infection induced autophagy in macrophages, and that butyrate further enhanced autophagy, increasing cytoplasmic p62 and LC3 levels. Moreover, butyrate reduced Keap1 levels in a dose-dependent manner. In contrast, *p62* knockdown and pharmacologic autophagy blockade prevented Keap1 degradation and caused its cytoplasmic accumulation. Collectively, these results demonstrate that butyrate promotes autophagy-mediated, p62-dependent Keap1 degradation.

Taken together, our results highlight an important role for butyrate in maintaining cellular redox homeostasis. These findings provide new insights into macrophage redox regulation and suggest potential therapeutic strategies for bacterial pneumonia. Despite these significant findings, our study has several limitations. For instance, we utilized PBMCs from clinical specimens for experimental analyses. However, PBMCs are not the primary target cells of *Klebsiella pneumoniae* infection (i.e., alveolar macrophages), and their functional properties differ substantially from those of alveolar macrophages. In future studies, we plan to collect bronchoalveolar lavage fluid from clinical patients, isolate alveolar macrophages for *in vitro* intervention assays, and directly evaluate the regulatory effects of butyrate on these pulmonary target cells. Additionally, we will explore the synergistic therapeutic potential of butyrate combined with conventional clinical antibiotics, to provide more comprehensive experimental evidence for its clinical translation. Furthermore, we observed that *K. pneumoniae* infection triggers 4-HNE production; however, the molecular targets underlying excessive accumulation of this oxidative byproduct and its associated pathological hazards warrant further in-depth mechanistic exploration.

## CRediT authorship contribution statement

**Bao Meng:** Writing – original draft. **Hongru Li:** Visualization. **Mingyang Tang:** Data curation. **Jie Zhu:** Formal analysis. **Ruixuan Yao:** Methodology. **Ying Xu:** Software. **Qingyue Zhang:** Formal analysis. **Rongqing Zhu:** Formal analysis. **Yaqin Guo:** Data curation. **Yuexin Xu:** Methodology. **Yuanlong Shu:** Software. **Liang Yu:** Investigation. **Yasheng Li:** Visualization. **Yi Yang:** Investigation. **Yanyan Liu:** Investigation. **Ting Wu:** Writing – review & editing. **Jiabin Li:** Writing – review & editing.

## Declaration of competing interest

All authors declare no known competing financial interests or personal relationships regarding the publication of this paper.
