# Supplementary material for: Butyrate protects against Klebsiella pneumoniae-induced oxidative stress in alveolar macrophages via p62-Keap1-Nrf2 pathway
Source: Redox Biol. 2026 Apr 12;93:104156. doi: 10.1016/j.redox.2026.104156 (PMC13094660; doi:10.1016/j.redox.2026.104156)
Supplement: Multimedia component 1 [file mmc1.docx]

**
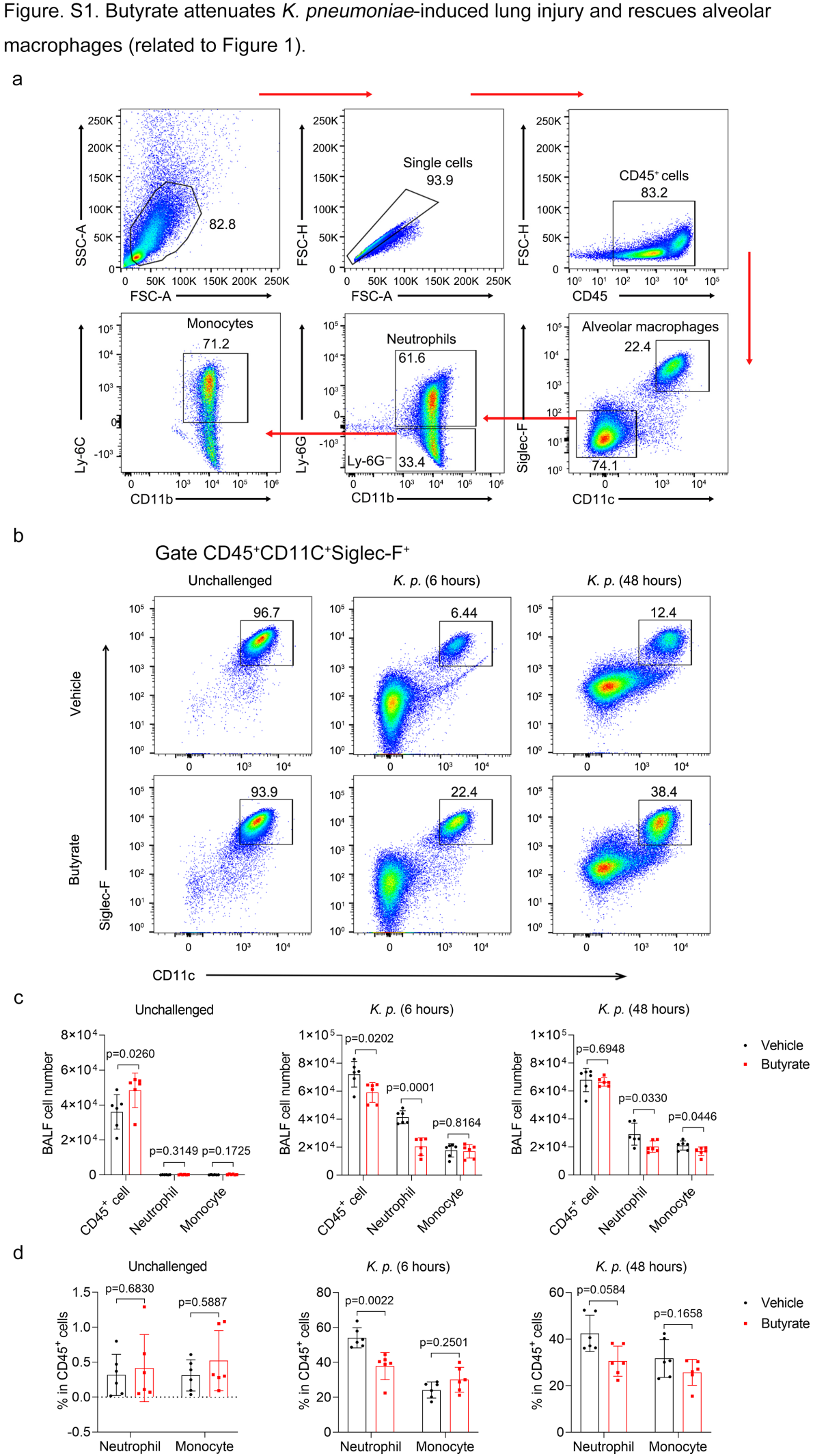
**

**Fig. S1. Butyrate attenuates *K. pneumoniae*-induced lung injury and rescues alveolar macrophages (related to Figure 1).**

1. Gating strategies for alveolar macrophages, neutrophils and monocytes used in Figure 1h and 7h.
2. Representative flow cytometry plots of alveolar macrophages from butyrate-treated and untreated mice at 6 and 48 hours post *K. pneumoniae* infection (n=6 per group).

(c) Total numbers of CD45^+^ cells, neutrophils and monocytes in BALF from butyrate-treated and untreated mice at 6 and 48 hours after *K. pneumoniae* infection (n=6 per group).

(d) Percentage of neutrophils and monocytes in CD45^+^ cells (n=6 per group).

All data are presented as mean ± SD. *p* values were determined by two-tailed unpaired Student's *t*-test (c, d).

**
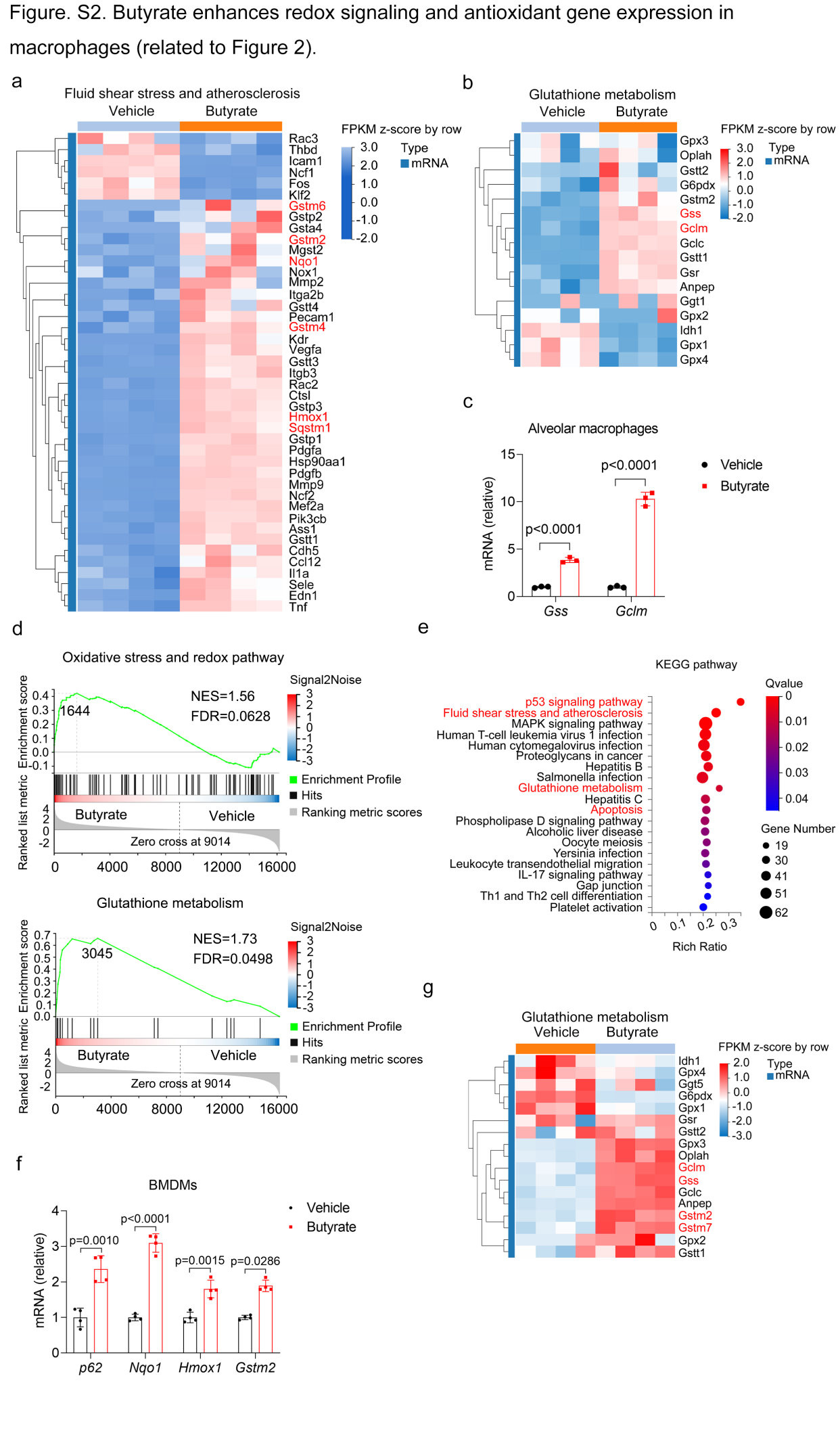
**

**Fig. S2. Butyrate enhances redox signaling and antioxidant gene expression in macrophages (related to Figure 2).**

(a-b) Hierarchical clustering heatmap of DEGs associated with the 'Fluid shear stress and atherosclerosis' and 'Glutathione metabolism'pathway.

(c) RT-qPCR analysis of *Gss* and *Gclm* expression in alveolar macrophages isolated from butyrate-treated and untreated mice (n=3 per group).

(d) GSEA of DEGs in butyrate-treated (1mM for 24 hours) and untreated BMDMs under *K. pneumoniae* challenge (n=4 per group).

(e) KEGG pathways enrichment analysis of DEGs. Top enriched pathways are highlighted in red.

(f) RT- qPCR analysis of p62 and antioxidant gene expression in BMDMs (n=4 per group).

(g) Hierarchical clustering heatmaps of DEGs related to the Glutathione metabolism.

All data are presented as mean ± SD. *p* values were determined by two-tailed unpaired Student's *t*-test (c, f). The DEGs were defined as those with *p* < 0.05 and fold change > 2. For GSEA, significance was defined as *p* < 0.05 and FDR *q*-value < 0.25.

**
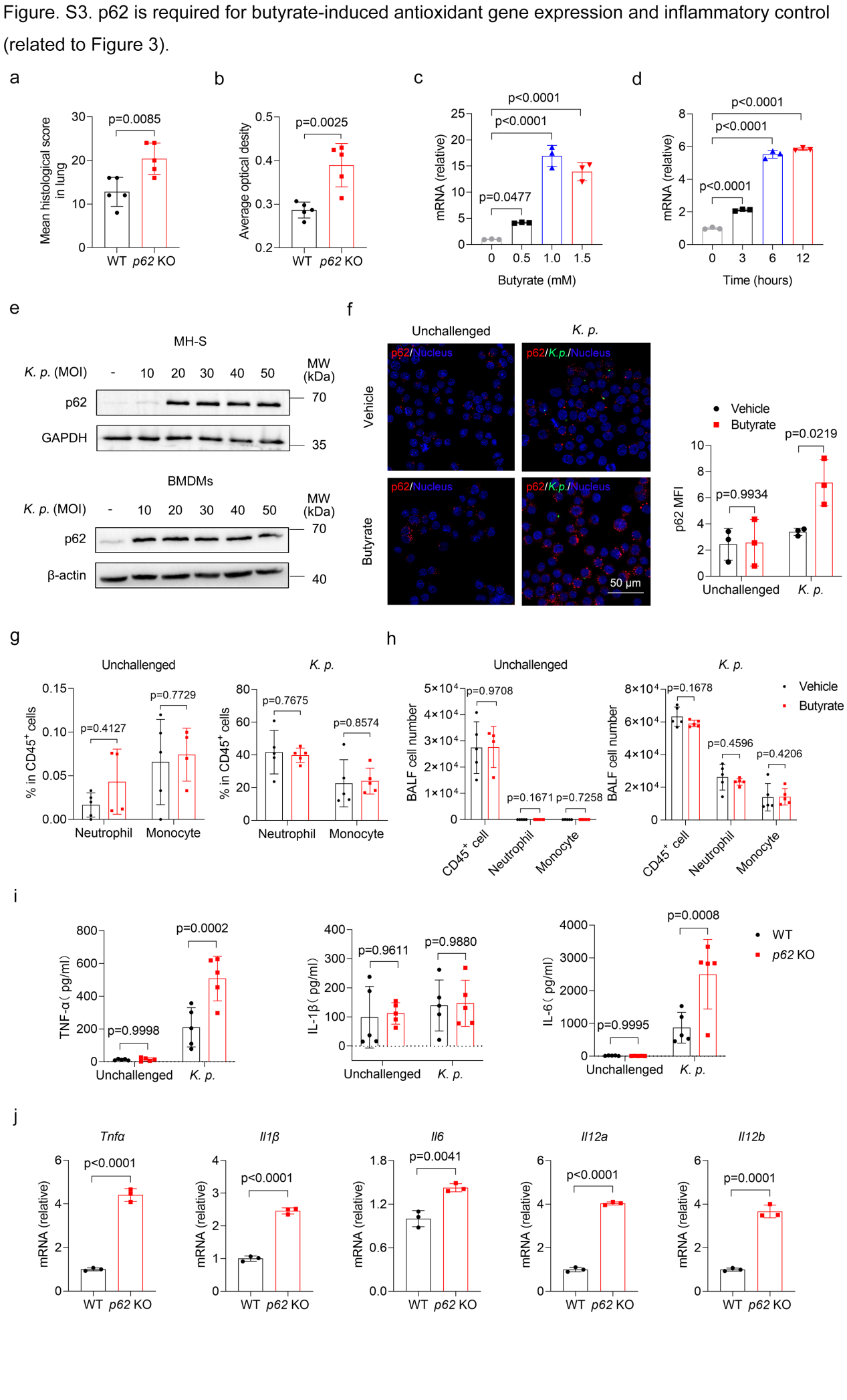
**

**Fig. S3. p62 is required for butyrate-induced antioxidant gene expression and inflammatory control (related to Figure 3).**

(a-b) Histological scoring and quantification of MPO immunohistochemical staining (n =5 per group).

(c-d) RT-qPCR analysis of *SQSTM1* expression in MH-S cells treated with varying concentrations or durations of butyrate under *K. pneumoniae* challenge (n =3 per group).

(e) Representative Western blot of p62 in MH-S cells and BMDMs challenged with *K. pneumoniae* at the indicated MOI.

(f) Representative immunofluorescence images and quantification analysis of the mean fluorescence intensity of p62 in butyrate-treated and untreated MH-S cells (n = 3 per group). Scale bars, 50 μm.

(g) Percentages of neutrophils and monocytes among CD45^+^ cells (n=5 per group).

(h) Numbers of CD45^+^ cells, neutrophils and monocytes in BALF from butyrate-treated and untreated *p62*^−/−^ mice at 6 hours post-infection with *K. pneumoniae* (n=5 per group).

(i) The levels of proinflammatory cytokines TNF-α, IL-6, and IL-1β in mice serum (n =5 per group).

(j) RT-qPCR analysis of indicated gene expression in *p62^+/+^* and *p62^-/-^* BMDMs following *K. pneumoniae* infection for 3 hours (n =3 per group).

All data are presented as mean ± SD. *p* values were determined by two-tailed unpaired Student's *t*-test (a-b, g-h, j), one-way ANOVA with Tukey's multiple-comparisons test (c-d) or two-way ANOVA (f, i).

**
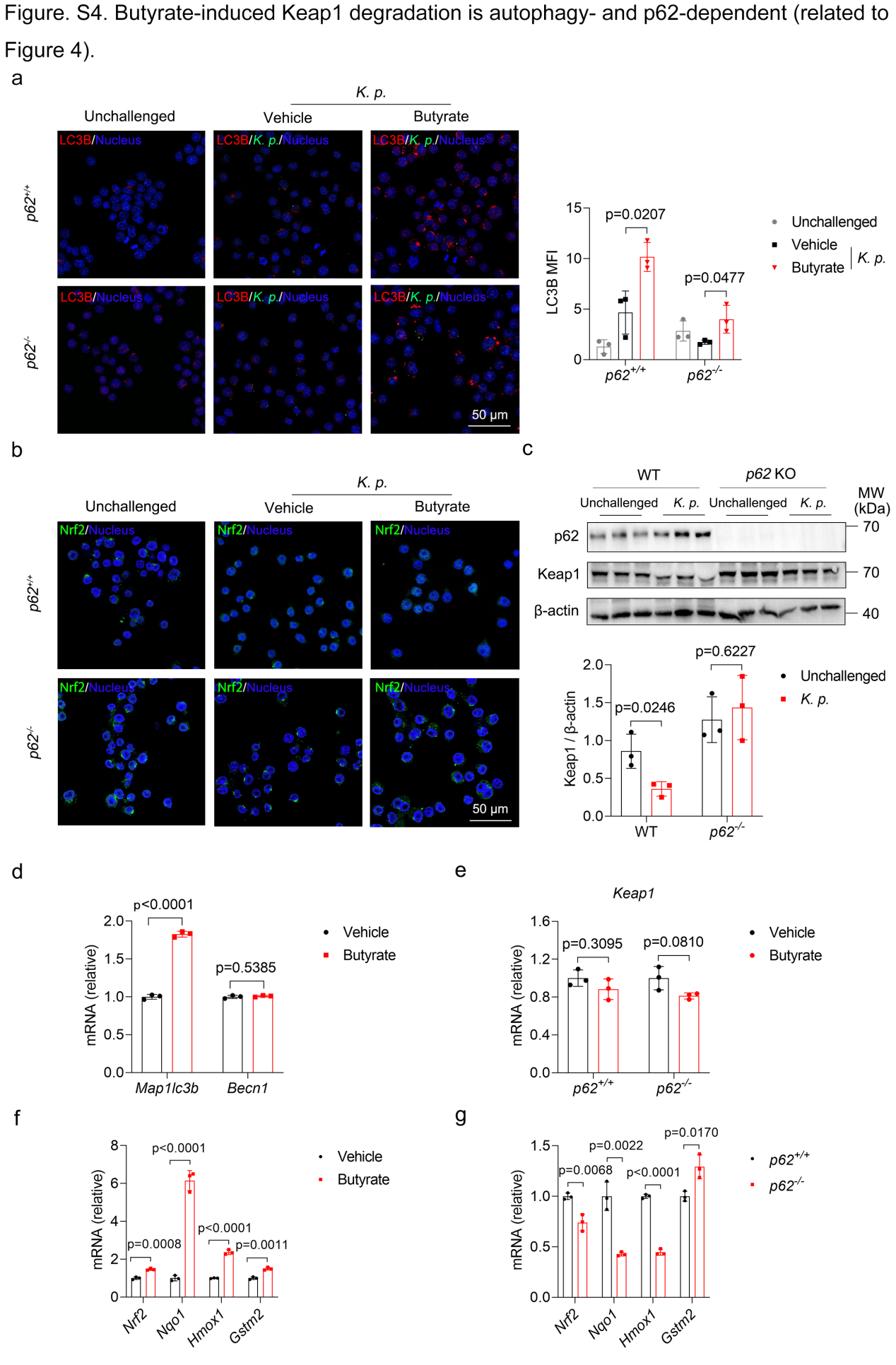
**

**Fig. S4. Butyrate-induced Keap1 degradation is autophagy- and p62-dependent (related to Figure 4).**

(a-b) Representative immunofluorescence images and quantification analysis of the mean fluorescence intensity of LC3B in butyrate-treated and untreated *p62^+/+^* or *p62^-/-^* MH-S cells following *K. pneumoniae* infection (n = 3 per group). Scale bars, 50 μm.

(b) Representative immunofluorescence images of Nrf2 from butyrate-treated and untreated *p62^+/+^* or *p62^-/-^* MH-S cells infected with *K. pneumoniae* for 3 hours (n = 3 for each group). Scale bars, 50 μm.

(c) Representative western blot of Keap1, p62 and densitometric quantification of Keap1 in lung tissues from *p62^+/+^* or *p62^-/-^* mice (n = 3 per group).

(d) RT-qPCR analysis of *Macp1lc3b* and *Becn1* in butyrate-treated and untreated MH-S cells infected with *K. pneumoniae* for 3 hours (n =3 per group).

(e) RT-qPCR analysis of *Keap1* in butyrate-treated and untreated *p62^+/+^* or *p62^-/-^* MH-S cells infected with *K. pneumoniae* for 3 hours (n = 3 for each group).

(f-g) RT-qPCR analysis of the indicated gene in butyrate-treated and untreated *p62^+/+^* or *p62^-/-^* MH-S cells infected with *K. pneumoniae* for 3 hours (n =3 per group).

All data are presented as mean ± SD. *p* values were determined using two-tailed unpaired Student's *t*-test (a, c, d, f-g) and two-way ANOVA (e).

**
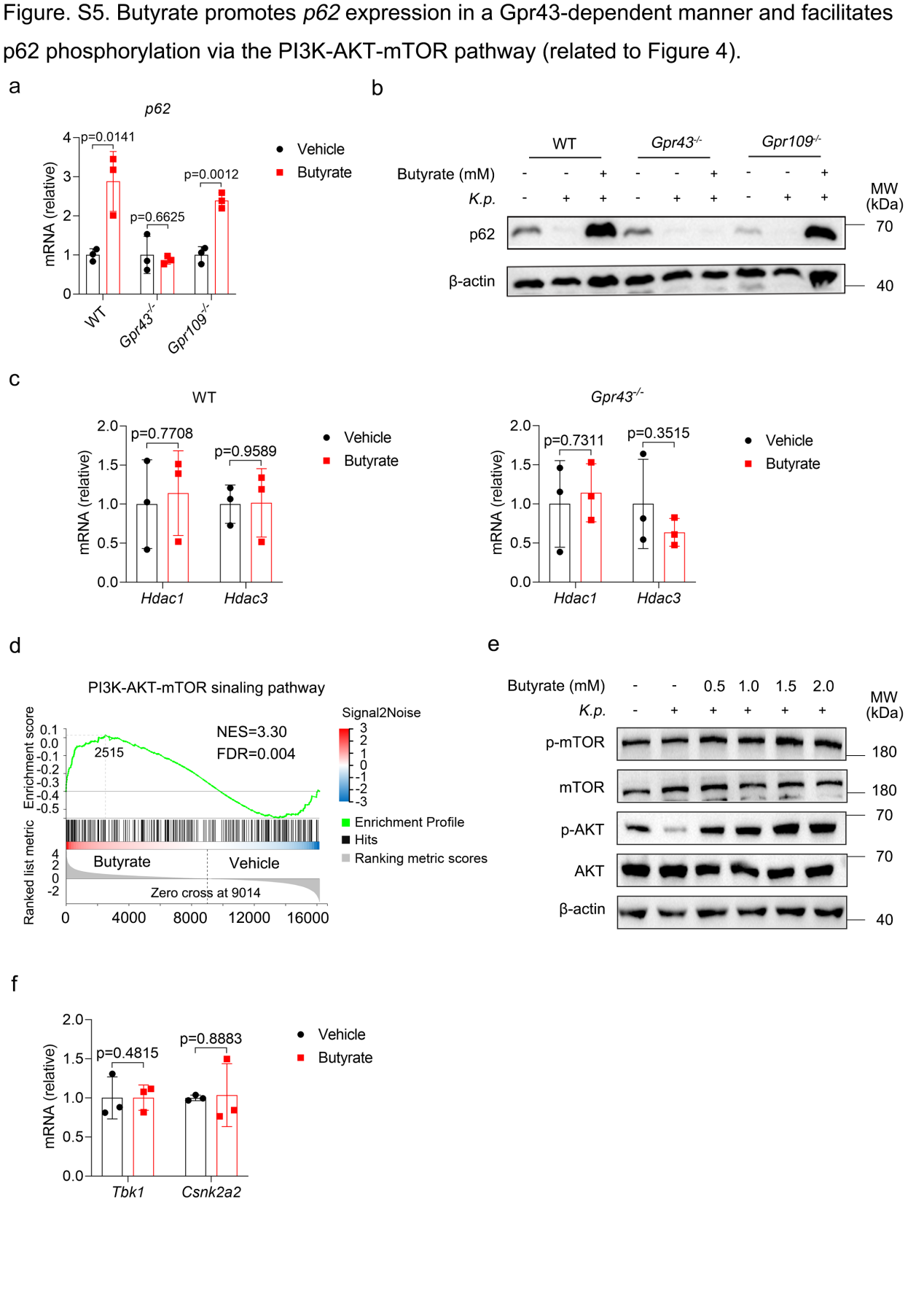
**

**Fig. S5. Butyrate promotes *p62* expression in a Gpr43-dependent manner and facilitates p62 phosphorylation via the PI3K-AKT-mTOR pathway (related to Figure 4).**

1. RT-qPCR analysis of *p62* in butyrate-treated and untreated BMDMs derived from WT, *Gpr43^-/-^* and *Gpr109a^-/-^* mice, following co-cultured with *K. pneumoniae* for 3 hours (n = 3 per group).
2. Representative western blot of p62 in BMDMs derived from WT, *Gpr43^-/-^* and *Gpr109a^-/-^* mice, treated with or without butyrate and then infected with *K. pneumoniae* for 3 h.
3. RT-qPCR analysis of *Hdac1* and *Hdac3* in butyrate-treated and untreated BMDMs derived from WT and *Gpr43^-/-^* mice, following co-cultured with *K. pneumoniae* for 3 hours (n = 3 per group).
4. GSEA of indicated DEGs in butyrate-treated versus untreated macrophages under *K. pneumoniae* infection.
5. Representative Western blot showing p-mTOR, mTOR, p-AKT and AKT in MH-S cells challenged with *K. pneumoniae* and treated with the indicated concentrations of butyrate.
6. RT-qPCR analysis of *Tbk1* and *Csnk2a2* in butyrate-treated and untreated MH-S cells infected with *K. pneumoniae* for 3 hours (n =3 per group).

All data are presented as mean ± SD. *p* values were determined using two-tailed unpaired Student's t-test (a, c, f).

**
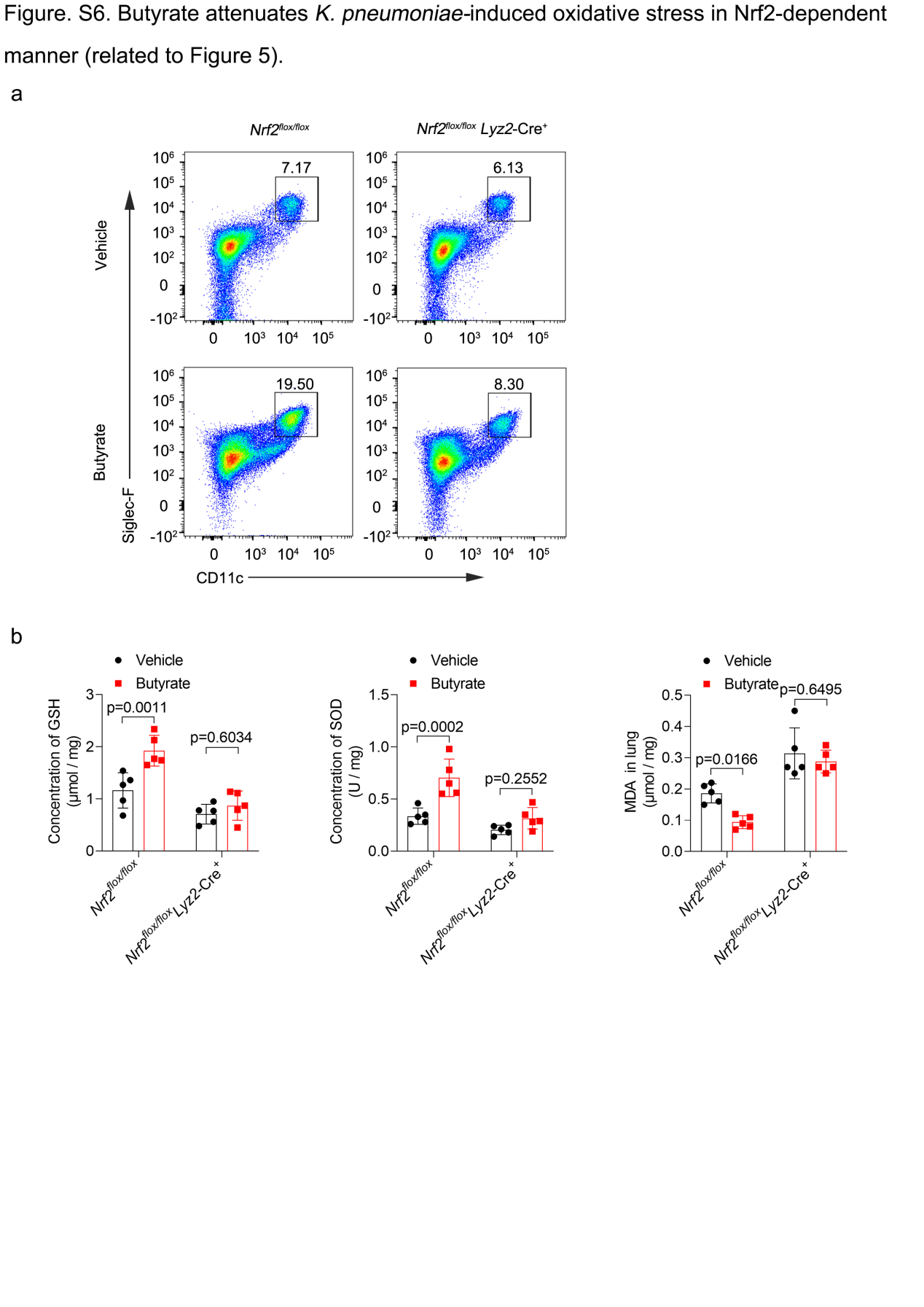
**

**Fig. S6. Butyrate attenuates *K. pneumoniae*-induced oxidative stress in Nrf2-dependent manner(related to Figure 5).**

1. Representative flow cytometry plots of alveolar macrophages from *Nrf2^flox/flox^* and *Nrf2^flox/flox^* *Lyz2*-Cre^+^ mice, either treated with butyrate or left untreated, at 6 h after *K. pneumoniae* infection (n = 5 per group).
2. Quantification analysis of MDA, SOD and GSH levels in lung tissues from *Nrf2^flox/flox^* and *Nrf2^flox/flox^* *Lyz2*-Cre^+^ mice treated with or without butyrate (n = 5 per group).

All data are presented as mean ± SD. *p* values were determined using two-way ANOVA (b).


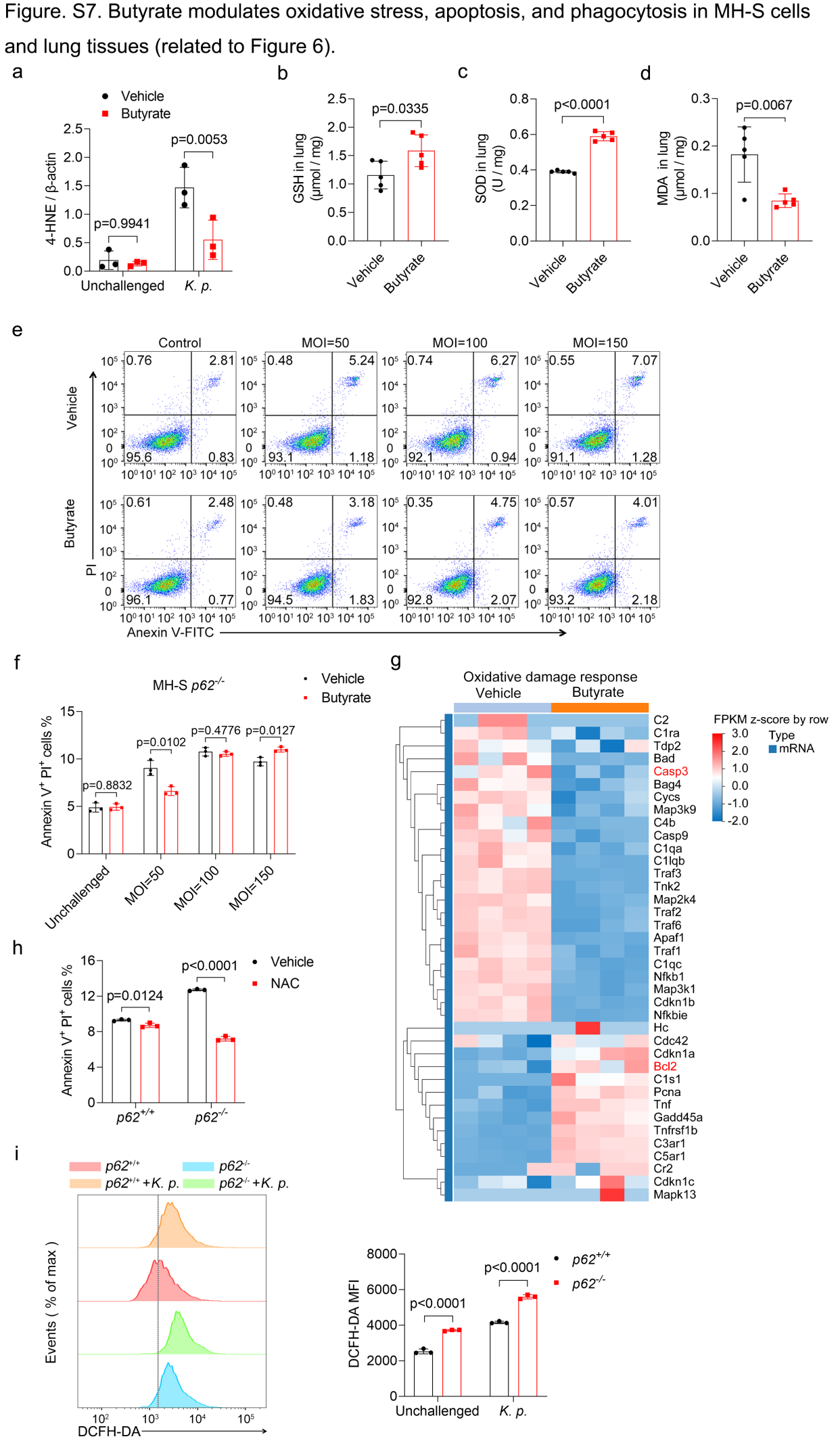


**Fig. S7. Butyrate modulates oxidative stress, apoptosis, and phagocytosis in MH-S cells and lung tissues (related to Figure 6).**

1. Representative Western blot densitometric quantification of 4-HNE in lung tissues from butyrate-treated and untreated mice (n=3 per group).

(b-d) Quantification analysis of MDA, SOD and GSH levels in lung tissues from butyrate-treated and untreated mice (n=5 per group).

(e) Representative flow cytometry plots of butyrate-treated and untreated MH-S cells infected with the indicated MOI of *K. pneumoniae* for 6 hours.

(f) Percentage of Annexin V^+^ / PI^+^ cells in *p62^-/-^* MH-S cells infected with the indicated MOI of *K. pneumoniae* for 6 hours (n = 3 per group).

(g) Hierarchical clustering heatmap of DEGs associated with the 'Oxidative damage response' pathway.

(h) Percentage of Annexin V^+^ / PI^+^ cells in NAC-treated MH-S cells infected with *K. pneumoniae* for 6 hours (n = 3 for each group).

1. Quantification of the intracellular ROS levels in *p62^⁺/⁺^* and *p62^-/-^* MH-S cells infected with *K. pneumoniae* for 1 hour (n = 3 per group).

All data are presented as mean ± SD. *p* values were determined using two-tailed unpaired Student's t-test (b-d, f) and two-way ANOVA with Tukey's multiple comparisons test (a, h, i).


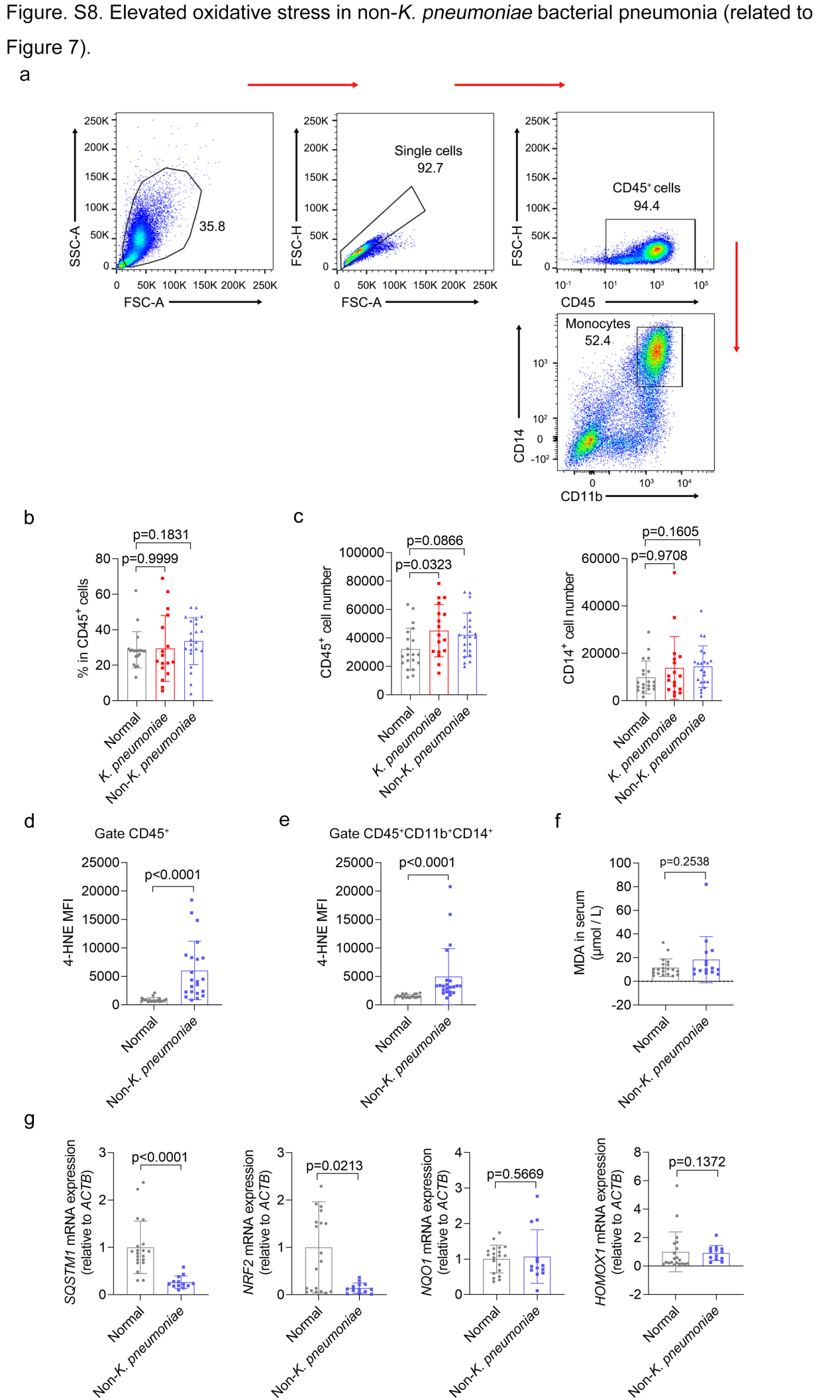


**Fig. S8. Elevated oxidative stress in non-*K. pneumoniae* bacterial pneumonia (related to Figure 7).**

(a) Gating strategies for CD45^+^CD11b^+^CD14^+^ monocytes used in Figure 5b.

(b-c) Total numbers of CD45^+^ cells, CD14^+^ monocytes in PBMCs from normal individuals (n=20) and patients with *Klebsiella* pneumonia (n=17) or non*-Klebsiella bacterial pneumonia* (n=22), and the percentage of CD14^+^ monocytes in CD45^+^ cells.

(d-e) Flow cytometric analysis of 4-HNE mean fluorescence in CD45^+^ cells and CD11B^+^CD14^+^ monocytes from normal individuals (n=20) and non*-Klebsiella bacterial pneumonia* patients (n=22).

(f) Concentration of MDA in serum from normal individuals (n=20) and patients with non*-Klebsiella bacterial pneumonia* (n=15).

(g) RT-qPCR analysis of *SQSTM1*, *NRF2, NQO1 and HOMOX1* mRNA expression in PBMCs from normal individuals (n=20) and patients with non*-Klebsiella bacterial pneumonia* (n=13).

All data are presented as mean ± SD. *p* values were determined using one-way ANOVA with Tukey's multiple-comparisons test (b-c), and two-tailed unpaired Student's *t*-test (d-g).

**
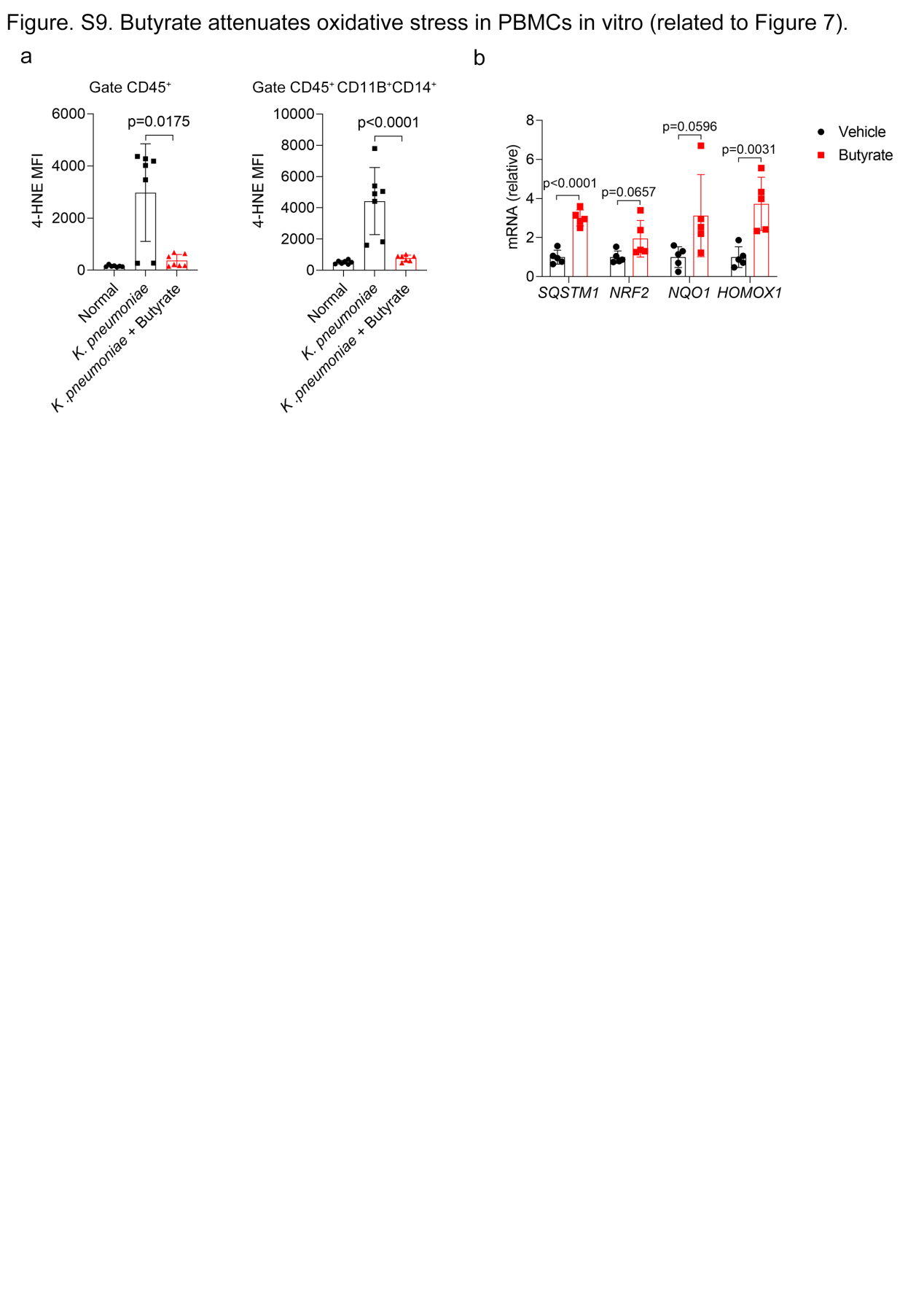
**

**Fig. S9. Butyrate attenuates oxidative stress in PBMCs in vitro (related to Figure 7).**

1. Flow cytometry analysis of 4-HNE levels in CD45⁺ cells and CD11B⁺CD14⁺ monocytes from PBMCs of healthy controls and patients with *Klebsiella* pneumoniae, with or without ex vivo butyrate treatment (n=7 per group).
2. RT-qPCR analysis of *SQSTM1*, *NRF2*, *NQO1*, and *HMOX1* mRNA expression in PBMCs from patients with *Klebsiella pneumoniae*, treated with or without butyrate (n=5 per group).

All data are presented as mean ± SD. *p* values were determined using two-tailed unpaired Student's *t*-test (a,b).


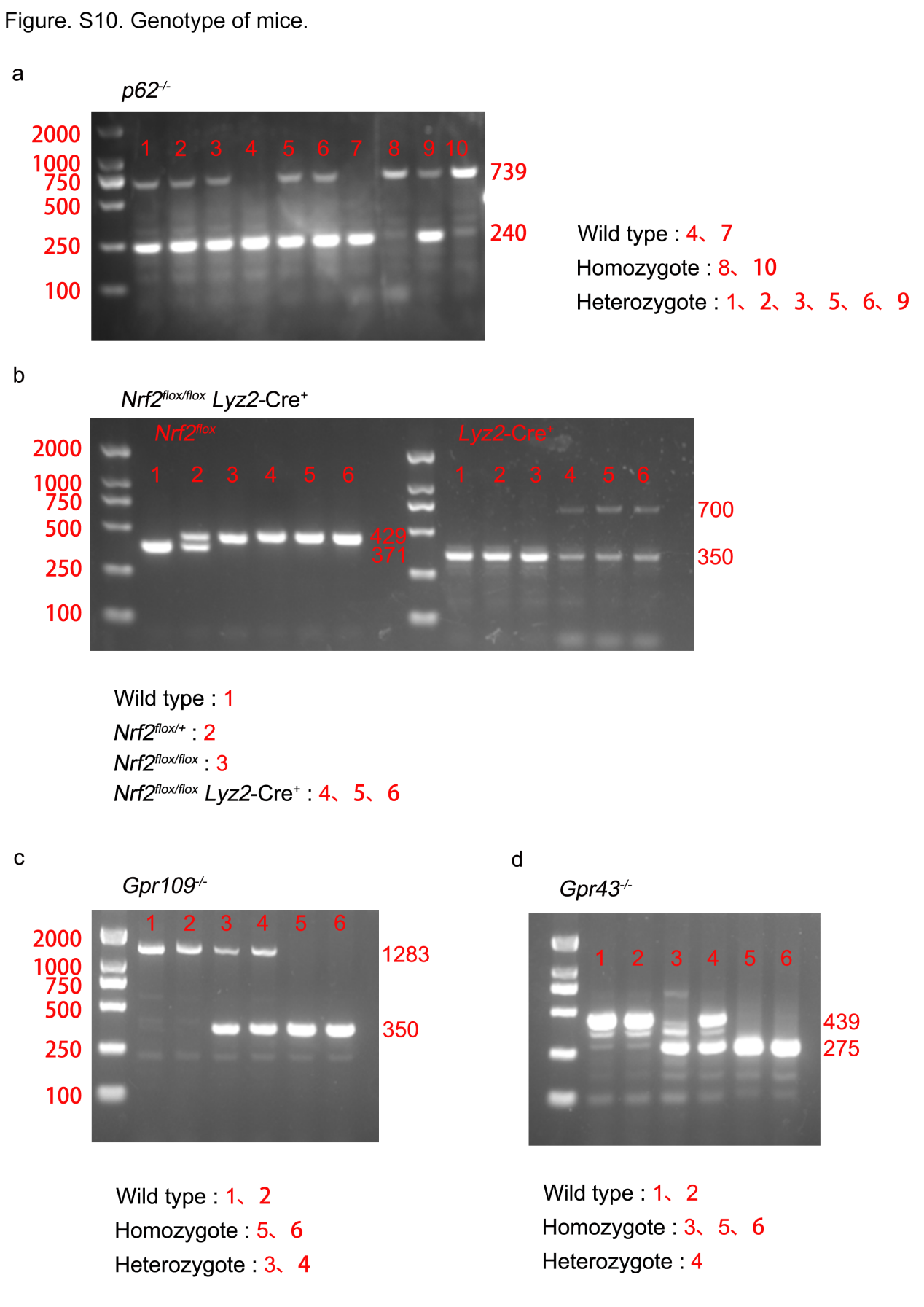


**Fig. S10. Agarose gel electrophoresis for genotyping of mice.**

1. Genotype of *p62* knockout.
2. Genotype of myeloid-specific conditional knockout of *Nrf2*.
3. Genotype of *Gpr109a* knockout.
4. Genotype of *Gpr43* knockout.
