# Supplementary material for: Butyrate protects against Klebsiella pneumoniae-induced oxidative stress in alveolar macrophages via p62-Keap1-Nrf2 pathway
Source: Redox Biol. 2026 Apr 12;93:104156. doi: 10.1016/j.redox.2026.104156 (PMC13094660; doi:10.1016/j.redox.2026.104156)

Figure. 2d

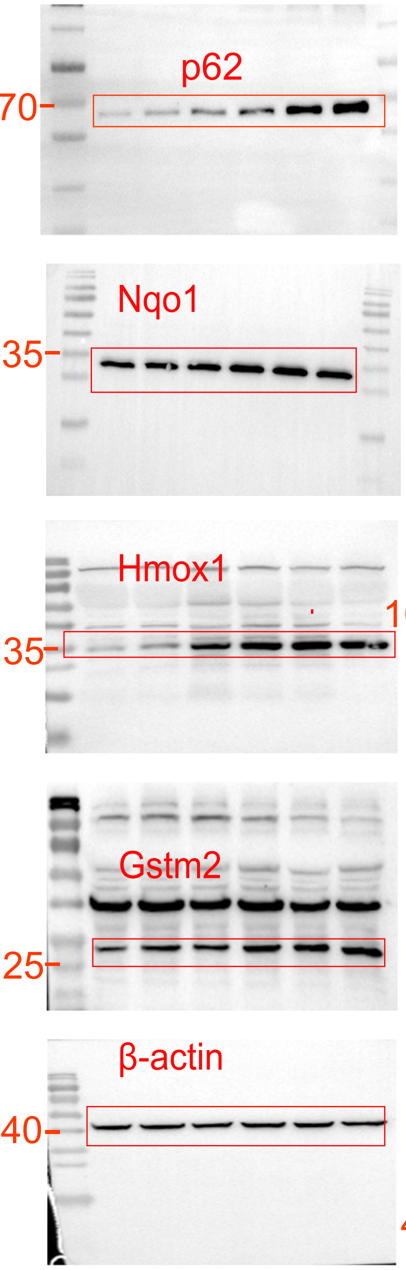

Figure. 4a

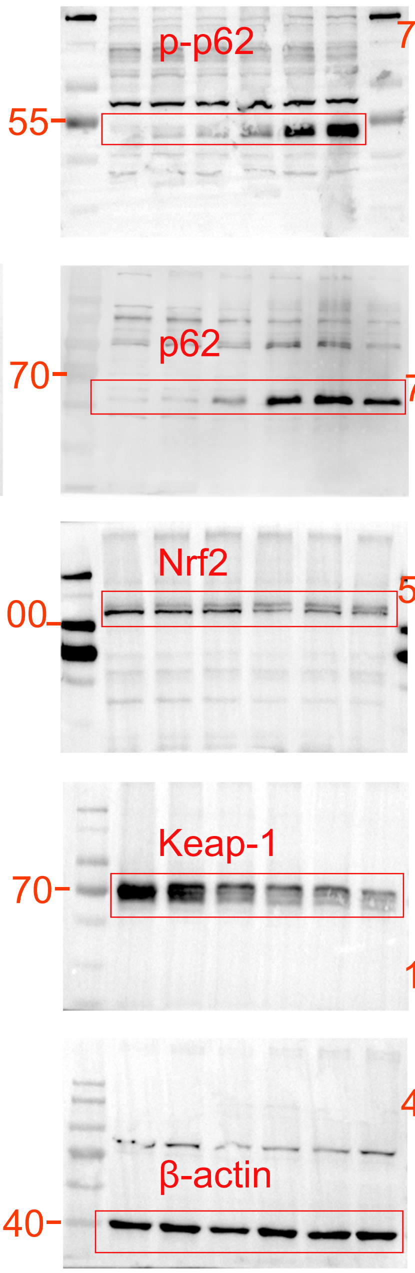

Figure. 4b

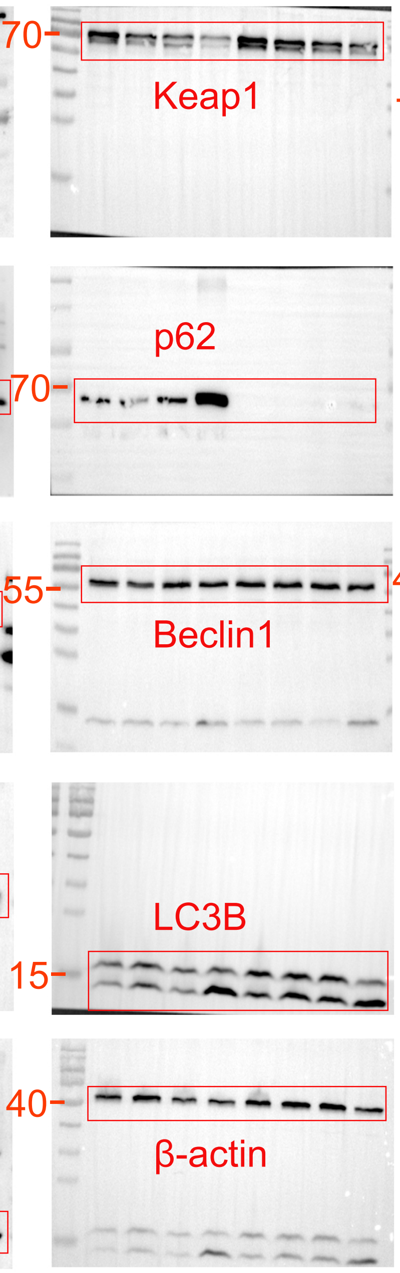

Figure. 4c

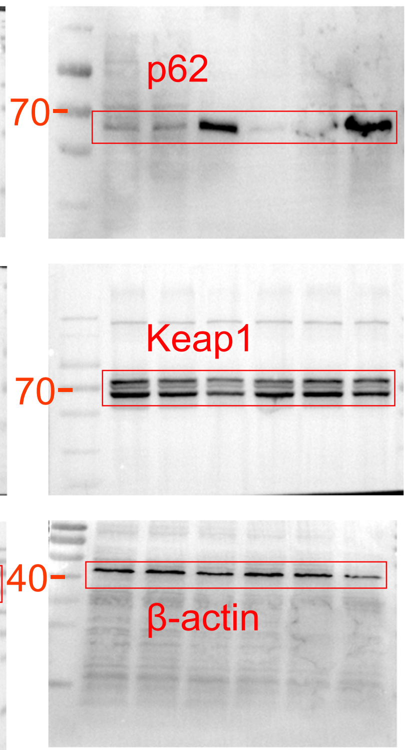

Figure. 3a

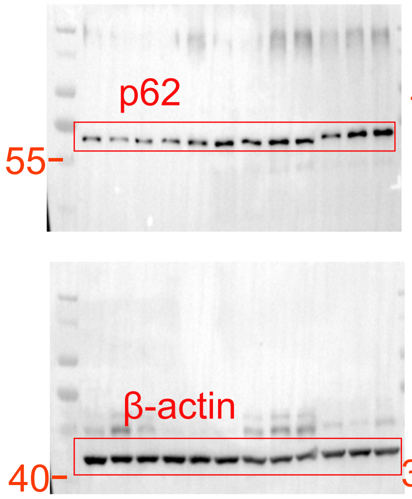

Figure. S3e
MH-S

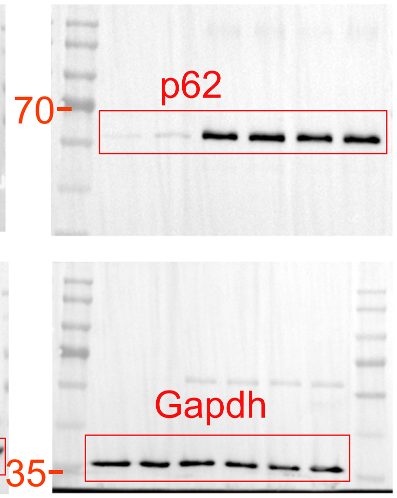

Figure. S3e
BMDMs

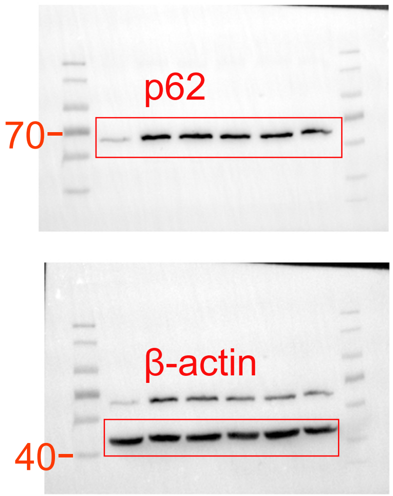

Figure. 4d

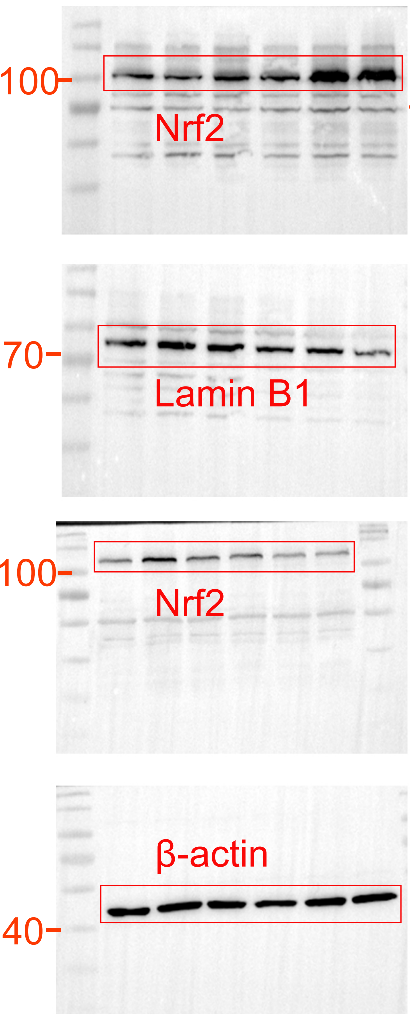

Figure. S4c

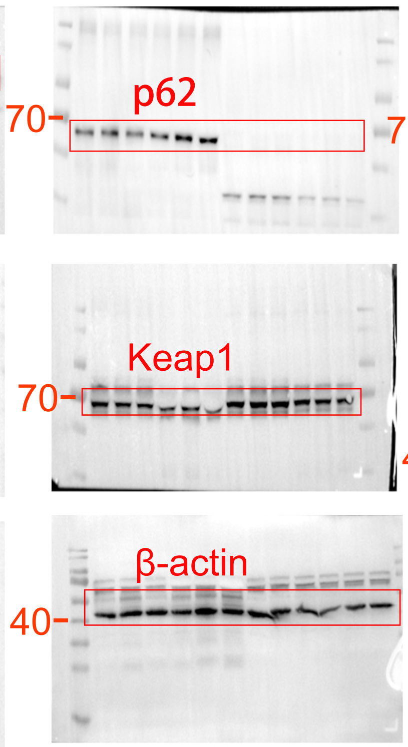

Figure. S5b

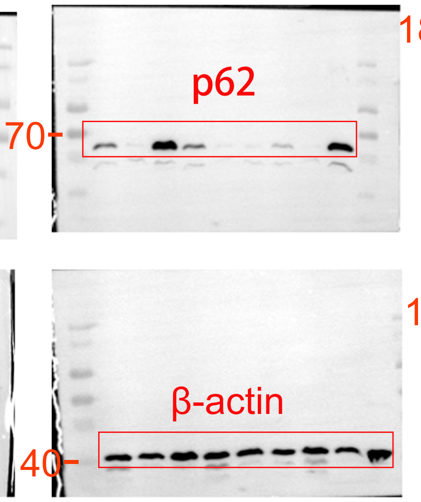

Figure. S5e

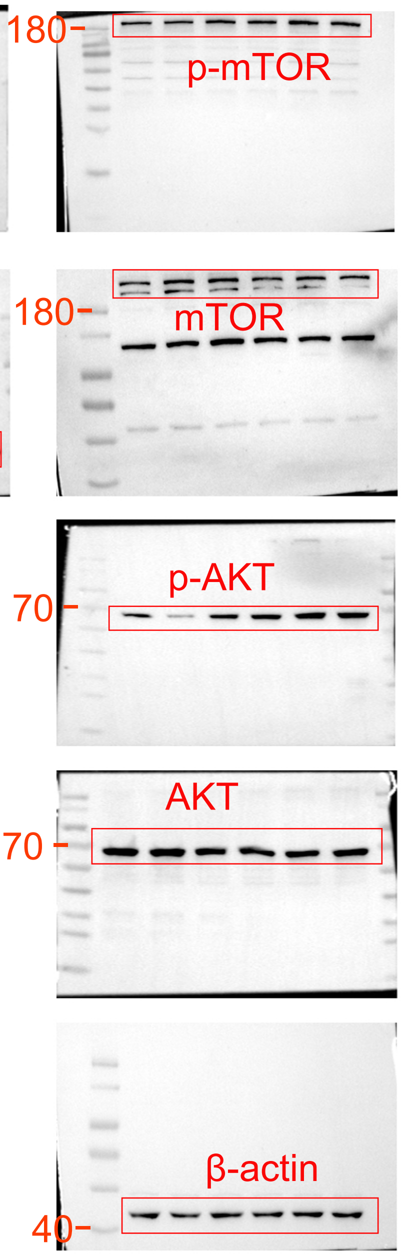

Figure. 6a

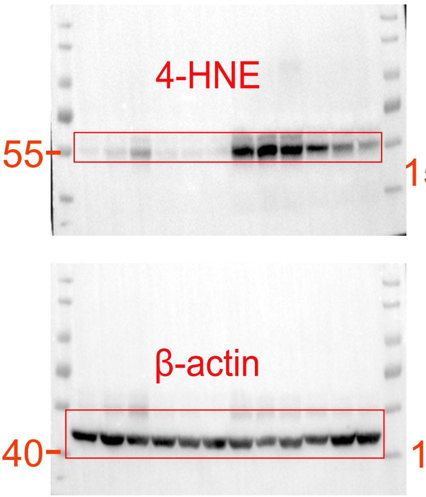

Figure. 6c

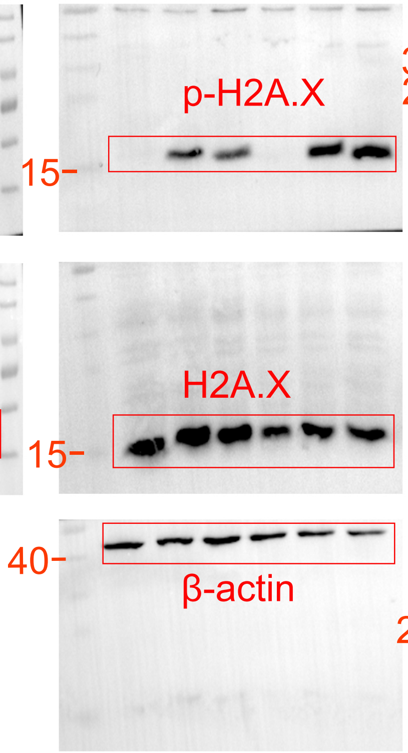

Figure. 6d

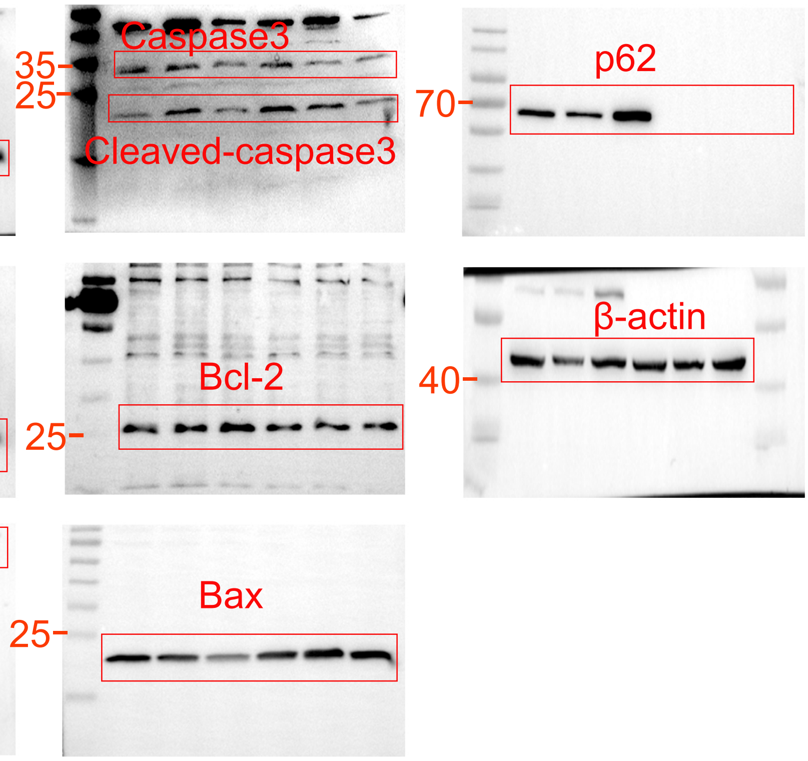

Supplement: Multimedia component 2 [file mmc2.pdf]
